# Targeting Kinases in *Fasciola hepatica*: Anthelminthic Effects and Tissue Distribution of Selected Kinase Inhibitors

**DOI:** 10.3389/fvets.2020.611270

**Published:** 2020-12-21

**Authors:** Carolin M. Morawietz, Hicham Houhou, Oliver Puckelwaldt, Laura Hehr, Domenic Dreisbach, Annika Mokosch, Elke Roeb, Martin Roderfeld, Bernhard Spengler, Simone Haeberlein

**Affiliations:** ^1^Institute of Inorganic and Analytical Chemistry, Justus Liebig University Giessen, Giessen, Germany; ^2^Biomedical Research Center Seltersberg (BFS), Institute of Parasitology, Justus Liebig University Giessen, Giessen, Germany; ^3^Department of Gastroenterology, Justus Liebig University Giessen, Giessen, Germany

**Keywords:** *Fasciola hepatica*, kinases, drug target, inhibitors, imatinib, BI 2536, MALDI mass spectrometry imaging, superoxide dismutase

## Abstract

Protein kinases have been discussed as promising druggable targets in various parasitic helminths. New drugs are also needed for control of fascioliasis, a food-borne trematode infection and worldwide spread zoonosis, caused by the liver fluke *Fasciola hepatica* and related species. In this study, we intended to move protein kinases more into the spotlight of *Fasciola* drug research and characterized the fasciolicidal activity of two small-molecule inhibitors from human cancer research: the Abelson tyrosine kinase (ABL-TK) inhibitor imatinib and the polo-like 1 (PLK1) inhibitor BI2536. BI2536 reduced viability of 4-week-old immature flukes *in vitro*, while adult worms showed a blockade of egg production. Together with a significantly higher transcriptional expression of PLK1 in adult compared to immature worms, this argues for a role of PLK1 in fluke reproduction. Both fluke stages expressed ABL1-TK transcripts at similar high levels and were affected by imatinib. To study the uptake kinetic and tissue distribution of imatinib in *F. hepatica*, we applied matrix-assisted laser desorption/ionization (MALDI) mass spectrometry imaging (MSI) for the first time in this parasite. Drug imaging revealed the accumulation of imatinib in different fluke tissues from 20 min to 12 h of exposure. Furthermore, we show that imatinib is metabolized to N-desmethyl imatinib by *F. hepatica*, a bioactive metabolite also found in humans. Besides the vitellarium, gastrodermal tissue showed strong signal intensities. *In situ* hybridization demonstrated the gastrodermal presence of *abl1* transcripts. Finally, we assessed transcriptional changes of physiologically important genes in imatinib-treated flukes. Moderately increased transcript levels of a gene encoding a multidrug resistance protein were detected, which may reflect an attempt to defend against imatinib. Increased expression levels of the cell cycle dependently expressed histone *h2b* and of two genes encoding superoxide dismutases (SODs) were also observed. In summary, our pilot study demonstrated cross-stage activity of imatinib but not BI2536 against immature and adult *F. hepatica in vitro*; a fast incorporation of imatinib within minutes, probably via the oral route; and imatinib-induced expression changes of physiologically relevant genes. We conclude that kinases are worth analyzing in more detail to evaluate the potential as therapeutic targets in *F. hepatica*.

## Introduction

Parasitic zoonoses have a dual impact by afflicting humans as well as animals, the latter causing substantial economic loss in livestock farming. Fascioliasis is a widely distributed zoonotic infection reported from almost half of all countries around the world, and it is caused by liver flukes of the genus *Fasciola* spp. (1). Herd-level prevalences reach up to 86% in some European countries and up to 91% in Africa (2, 3). Not surprisingly, the global economic losses in animal milk and meat production were estimated at several billion $/year (2). Besides animals, *Fasciola* spp. infects about 2.4–17 million humans (1). The WHO classifies fascioliasis as a neglected tropical disease (NTD), because particularly resource-poor countries are afflicted and the common impetus for disease control is insufficient. While fascioliasis proceeds subclinically in most patients, it can also result in anemia, weight loss, malnutrition, and biliary cirrhosis, which decrease quality of life and cause devastating developmental deficits in children and adults (4). Under the One Health aspect, finding effective control measures for both human and livestock infections appears obligatory. Among the most important species with respect to human and animal health is *Fasciola hepatica*, which infects its final host by metacercariae. Upon oral ingestion, juvenile flukes hatch in the small intestine and penetrate the intestinal wall to reach the liver. After penetrating the liver capsule, the growing and maturing liver flukes migrate, and feed through the liver parenchyma during the acute stage of infection. As adults, they reside in the bile ducts and cause the chronic stage of disease (5). Triclabendazole is the drug of choice because of its activity against both immature and adult fluke stages. Probably as a consequence of its massive use in the livestock industry, triclabendazole-resistant *F. hepatica* strains have spread in numerous countries, which motivates the search for alternative treatment options (6).

Protein kinases (PK) have been discussed as druggable targets with high potential in various parasitic helminths, including cestodes, trematodes, and filaria (7–9). In mammalian cells, PK are known as regulators of fundamental biological processes, including cell cycle control and cell differentiation (10, 11). Employing PK as targets is even more attractive, as numerous small-molecule kinase inhibitors are at hand that have been pursued as anti-cancer drugs. This allows to piggy-back on existing drugs, which could potentially be repurposed or serve as a basis for the design of optimized anthelmintic kinase inhibitors (12). While kinase inhibitors have been intensely tested against blood flukes (*Schistosoma* spp.), the study of kinases as targets has been largely neglected in the related liver flukes. Previous studies focused on the usefulness of selected kinases as diagnostic marker for *Fasciola* spp. (phosphoenolpyruvate carboxykinase) or as vaccine candidate (phosphoglycerate kinase) (13, 14). The presumably only kinase evaluated as chemotherapeutic target is phosphofructokinase and dates back to 1962 (15, 16). However, work on a related phosphofructokinase inhibitor was not further continued because of suboptimal *in vivo* efficacy (17).

To move kinases more into the spotlight of *Fasciola* drug research, we recently demonstrated a cross-stage expression pattern of several kinases that have been described as anthelminthic targets in other species (18). Targeting several fluke stages by the same kinase inhibitor thus appears achievable. Here, we extended these studies and evaluated the fasciolicidal effects of two kinase inhibitors with well-described antischistosomal activity: the Abelson tyrosine kinase (ABL-TK) inhibitor imatinib and the polo-like kinase (PLK) inhibitor BI2536 (19–21). Biochemical studies confirmed that schistosomal ABL-TK and PLK1 are targets for imatinib and BI2536, respectively (22–24). We previously identified potential orthologs of ABL-TK and PLK1 kinases in *F. hepatica* (18), which makes imatinib and BI2536 excellent candidates for a pilot study of kinase inhibitors with fasciolicidal potential.

Next to quantifying *in vitro* effects of imatinib and BI2536 on the viability of two pathogenic stages of *F. hepatica*, we studied the tissue distribution of imatinib in adult flukes. To this end, we applied matrix-assisted laser desorption/ionization (MALDI) mass spectrometry imaging (MSI) for the first time in this parasite. Drug imaging by this technique allowed us to answer fundamental questions such as “How fast is imatinib taken up by the fluke?,” “In which tissues does it accumulate?,” and “Is it metabolized?” Finally, we assessed transcriptional changes of genes associated with oxidative stress, cell cycle, and drug responses in imatinib-treated flukes, which allowed us to speculate on a possible anthelmintic mode of action.

## Materials and Methods

### Ethical Statement

Rats (*Rattus norvegicus*) were used as model hosts in accordance with the European Convention for the Protection of Vertebrate Animals used for Experimental and Other Scientific Purposes (ETS No 123; revised Appendix A). The experiments were approved by the Regional Council (Regierungspraesidium) Giessen (V54-19c20 15 h 02 GI 18/10 Nr. A16/2018).

### Harvesting of *F. hepatica*

Male Wistar rats RjHan:WI (Janvier, France) served as final host to obtain immature and adult stages of *F. hepatica*. Rats at 5 weeks age were orally infected with 25 metacercariae from an Italian strain of *F. hepatica* (Ridgeway Research, UK). Immature flukes were collected from livers at 4 weeks p.i., and adult flukes were collected from bile ducts at 12 weeks p.i. Worms were kept for 1 h in 0.9% NaCl to allow clearance of gut contents. Parasites were then used for *in vitro* culture experiments, or they were embedded in Tissue-Tek (Sakura Finetek, The Netherlands) for subsequent *in situ* hybridizations.

### *In vitro* Culture and Inhibitor Treatment

The anthelminthic activity of selected kinase inhibitors against immature and adult stages of *F. hepatica* was assessed *in vitro*. The worms were individually cultured in 12-well-plates in RPMI medium supplemented with 5% chicken serum, 1% ABAM-solution (10,000 units penicillin, 10 mg streptomycin, and 25 mg amphotericin B per milliliter) (all from Gibco), and 100 μg/ml gentamycin (Sigma-Aldrich, USA). Different concentrations of a kinase inhibitor (20, 50, 100, or 150 μM) or the same volume of the inhibitor's solvent dimethyl sulfoxide (DMSO) was added as a negative control. The following kinase inhibitors were tested: the PLK1 inhibitor BI2536 (Selleckchem, Germany) and the ABL-TK inhibitor imatinib [imatinib mesylate, purity ≥ 98% (HPLC); Enzo Life Sciences, Germany]. The flukes were incubated at 37°C in a 5% CO_2_ atmosphere for 72 h, and medium plus inhibitor was refreshed every 24 h. Inhibitor-induced effects on worm viability were assessed every 24 h using a stereo microscope at 10× magnification (M125 C, Leica, Germany). Worm motility was assessed using the following scores: 3 (normal motility), 2 (reduced motility), 1 (minimal and sporadic movements), and 0 (no movement even upon mechanical stimulation with forceps was considered dead). When needed, egg numbers produced in the last 24 h of the 72-h culture period were counted. After the 72-h culture, flukes were immersed in Monarch RNA Protect Buffer (New England BioLabs, USA), individually snap frozen in liquid nitrogen, and stored at −80°C until RNA extraction.

### MTT Assay

The hepatocyte cell line FL83B (25) was used to study the cytotoxic effects of imatinib. The same cell line was used to quantify cytotoxicity of triclabendazole as a reference compound to allow a direct comparison of the cytotoxic potential of both compounds. FL83B cells were obtained from ATCC (#CRL-2390) and cultured as recommended by ATCC. FL83B cells were exposed to imatinib and triclabendazole (analytical standard; Sigma). Imatinib was dissolved in pure water while triclabendazole was dissolved in DMSO. Initial cell densities of 25,000–50,000 cells/well (96 well-plate, Corning, Costar, flat bottom) turned out to be in the optimum range for all of the tests and cells were allowed to settle for 24 h. Cell viability tests were performed after 24 h of exposure to the test substances. Subsequently after removal of the medium containing test substances, cells were incubated for 1 h with 0.5 mg/ml MTT (Sigma # M5655) in culture medium for all assays. After the incubation with MTT and removal of the medium, 100 μl of DMSO was added and plates were incubated for 1 h. Absorption of formazan was measured at 570 nm using a microplate reader (Tecan Infinite M Plex, Tecan, Austria). The cytotoxicity was calculated as % dead cells (26):

$$\begin{array}{l} 100\text{\%}-[({\text{Absorbance in test}}^{*}\text{ wells}/{\text{Absorbance in control}}^{**}\\ \text{wells})/\times 100U] \end{array}$$

^*^Test wells were the test-substance containing wells. ^**^Control wells were wells without test substance, i.e., 100% survival. EC50 value was calculated in Excel by plotting log(conc.) on the ordinate against cytotoxicity (abscissa). An optimal trendline was assessed by polynomial fitting and subsequently used for calculation of the EC50 values. Non-linear least-squares data fitting was used to visualize the cytotoxicity plot (27). All tests were performed at least two times and each time in duplicate.

### Identification of Orthologous Genes

Orthologs of genes were identified in the genome of *F. hepatica* (Center for Genomic Research, University of Liverpool, BioProject ID PRJEB25283, version 11.0 of August 2018) using BLASTp searches starting from known orthologs in *S. mansoni* and using the public domain tool WormBase ParaSite, version WBPS13 (https://parasite.wormbase.org) (28). Correct annotation of the hits was further confirmed by another BLASTp search in NCBI against the genome of *H. sapiens*. Gene names, accession numbers, and biological function are listed in Supplementary Table 1. The identity of the potential *F. hepatica* orthologs was further verified by confirming the presence of conserved protein domains using SMART (http://smart.embl-heidelberg.de/) (29). Transmembrane helices in proteins were predicted using the membrane protein topology prediction method TMHMM (Server v. 2.0), based on a hidden Markov model (http://www.cbs.dtu.dk/services/TMHMM/).

### RNA Isolation and cDNA Synthesis

Total RNA from immature and adult flukes was extracted using the Monarch total RNA Miniprep kit (New England BioLabs) following the manufacturer's protocol. Individual flukes were stored and then mechanically homogenized in 300–600 μl of 1× RNA/DNA protection buffer. RNA quality and quantity were assessed using the BioAnalyzer 2100 and an Agilent RNA 6000 Nano or Pico Chip according to the manufacturer's instructions (Agilent Technologies, USA). cDNA was synthesized from 5 to 10 ng of total RNA using the QuantiTect Reverse Transcription Kit (QIAGEN, Germany) including a genomic DNA removal step. A 1:5–1:10 dilution of cDNA was used as template in quantitative real-time PCR (qRT-PCR).

### Quantitative Real-Time PCR

Gene expression was determined in adult flukes after inhibitor treatment using qRT-PCR. Only living flukes were used for analyses. Primers were commercially synthesized by Integrated DNA Technologies IDT (USA) and designed for an amplicon size of 141–215 bp and a melting temperature of 60°C (Supplementary Table 2) using the Primer3Plus software tool (30). When possible, primer pairs were located on different exons of a gene to exclude amplification of contaminating genomic DNA. All primer pairs yielded one specific PCR product, no primer dimers, and an amplification efficiency of 85–100%. qRT-PCRs were run on a Rotorgene Q cycler (QIAGEN, Germany) using the 2× PerfeCTa SYBR Green SuperMix (Quantabio, USA) in a final volume of 10 μl and 400 nM of each primer. The following PCR conditions were applied: initial denaturation step at 95°C for 3 min, 45 cycles at 95°C for 10 s, 60°C for 15 s, and 72°C for 20 s. Melting curve analysis was performed to exclude the generation of primer dimers and to verify primer specificity. All qRT-PCRs were run with three technical replicates and mostly comprised three to five biological replicates. The expression of genes of interest was determined by relative quantification against the geometric mean of two reference genes (glutamyl-prolyl-tRNA synthetase, Fh*eprs*, and tubulin-specific chaperone D, Fh*tbcd*) (18). Relative expression levels were expressed as n-fold difference vs. the expression in control flukes based on the ΔΔCt method (relative expression = 2^−ΔΔCt^). For the quantification of kinase transcript levels, relative expression values were calculated by the formula: relative expression = 2^−ΔCt^ × *f*, with *f* = 1,000 as an arbitrary factor.

### *In situ* Hybridization

To detect the occurrence of transcripts of Fh*abl1, in situ* hybridization was performed as described earlier with slight modifications (31). Twelve-week-old *F. hepatica* worms were embedded in Tissue-Tek (Sakura Finetek), frozen on dry ice, and stored at −80°C until use. Traversal cryosections of 10 μm thickness were prepared using a cryostat HM525 (Thermo Fisher Scientific, Germany). Sections were dried, post-fixed in 4% PFA, and permeabilized with PBSTx. The hybridization reaction was carried out overnight at 55°C. Following washing with saline sodium citrate buffer (SSC), the sections were incubated with anti-DIG antibodies coupled with alkaline phosphatase (Roche, Germany). After subsequent washing steps with maleic acid buffer with Tween (MAB-T), the development reaction was carried out using naphthol-AS-phosphate, and Fast Red TR (Sigma). The Riboprobes for the hybridization reaction were generated as previously described (32). The following primers were used to generate the template for probe synthesis with a length of 596 bp: 5′-GAATCTCCTTCTCCTAACGGT-3′, reverse 5′-ACCAGATTTTTTAGGAGGTCTTC-3′. The labeling reaction using digoxigenin-11-UTP (NU-803-DIGXS; Jena Bioscience, Germany) was done using T3 or SP6 RNA polymerases for sense and antisense probes. Labeled transcripts were controlled for the correct size by gel electrophoresis.

### Embedding of *F. hepatica* for AP-SMALDI MSI

Imatinib-treated adult flukes were used for drug imaging by atmospheric-pressure scanning microprobe MALDI MSI (AP-SMALDI MSI). After treatment with 100 μM imatinib for different time periods (20 min, 4 h, and 12 h), the worms were quickly immersed in PBS followed by distilled water. Subsequently, they were embedded in 8 wt% aqueous gelatin solution (gelatin powder, VWR, USA) using Tissue-Tek cryomolds (15 × 15 × 5 mm^3^) (Sakura Finetek), frozen on dry ice, and stored at −80°C until use. Transversal sections of embedded worms of 20 μm thickness were obtained using a cryostat HM525 (Thermo Fisher Scientific) at around −23°C. The quality of the sections was monitored by a digital light microscope (VHX-5000; Keyence, Japan) to obtain optical images in 250× magnification. Sections were stored at −80°C until AP-SMALDI MSI sample preparation. For each timepoint of imatinib treatment, two flukes were analyzed.

### AP-SMALDI MSI Sample Preparation and Measurements

Before matrix application, tissue sections were defrosted and protected from humidity for 10 min in a desiccator. A matrix solution consisting of 2,5-dihydroxybenzoic acid (DHB for synthesis, Merck, Germany) in a concentration of β = 30 g/L solved in acetone/water (acetone uvasol, Merck; water HiPerSolv Chromanorm for HPLC, filtered at 0.2 μm, VWR) 1:1 *v*/*v* with 0.1 vol% trifluoroacetic acid (TFA, uvasol for spectroscopy, Merck) was freshly prepared before measurement. A volume of 100 μl of matrix solution was sprayed onto the sample surface using an ultrafine pneumatic sprayer system (SMALDIPrep, TransMIT GmbH, Germany) with a flow rate of 10 μl/min and N_2_-pressure of 1 bar. AP-SMALDI MSI was performed on a Q Exactive orbital trapping mass spectrometer (33) (Thermo Fisher Scientific) equipped with an autofocusing AP-SMALDI5 AF ion source (34) (TransMIT GmbH). Measurements were conducted with a step size of 10 μm under activation of the pixel-wise autofocusing feature, and 50 laser pulses were applied per pixel. All imaging experiments were performed in positive-ion mode in *m*/*z* range of 250–1,000 with a mass resolution of 140,000 at *m*/*z* 200. For internal calibration, the lock masses *m*/*z* 273.03937 (corresponding to [2DHB+H−2H_2_O]^+^) and *m*/*z* 716.12462 (corresponding to [5DHB−4H_2_O+NH_4_]^+^) were set. Further adjusted parameters were a maximum ion injection time of 500 ms, an s-lens level of 100.0 arbitrary units, 300°C capillary temperature, as well as an acceleration voltage of 3.00 kV.

### Data Acquisition and Analysis of AP-SMALDI MSI Experiments

For data acquisition, Q Exactive Tune (version 2.9, Thermo Fisher Scientific) was used, and the ion source was operated by the SMALDIControl software (V1.1-118, TransMIT GmbH). XCalibur (version 4.0.27.13, Thermo Fisher Scientific) was applied for processing of mass spectra. Mirion software package (version 3.2.64.16) (35) (TransMIT GmbH) was utilized for visualization of imaging data; for data evaluation, the histogram bin width was adjusted to 0.005 u. No TIC normalization was used for image creation. Compounds were assigned based on accurate mass measurements with a mass tolerance of <3 ppm and LIPIDMAPS (36) database searches. For further evaluation, Root-mean-square-error (RMSE) plots were created using the Mirion software package.

### H&E-Staining After AP-SMALDI MSI

After imaging, the matrix layer was washed off with 80 vol% aqueous ethanol (EtOH purissimum, Roth, Germany) and stained according to the hematoxylin and eosin (H&E) staining protocol (Mayer's Hematoxylin and Eosin-Y solution, Sigma-Aldrich; m- and p-Xylol for analysis, Merck; Eukitt quick hardening mounting medium for microscopy, Honeywell-Fluka, USA). Optical images (250 × magnification) were recorded after the mounting medium has dried, using a digital light microscope (VHX-5000, Keyence).

### Statistical Analysis

Statistical significance was tested using the non-parametric Wilcoxon rank sum test (https://ccb-compute2.cs.uni-saarland.de/wtest/) (37). *p* <0.05 was considered statistically significant. Error bars represent the standard error of the mean (SEM).

## Results

### Transcriptional Expression of Selected Kinases in Pathogenic Stages of *F. hepatica*

**Figure 1:**
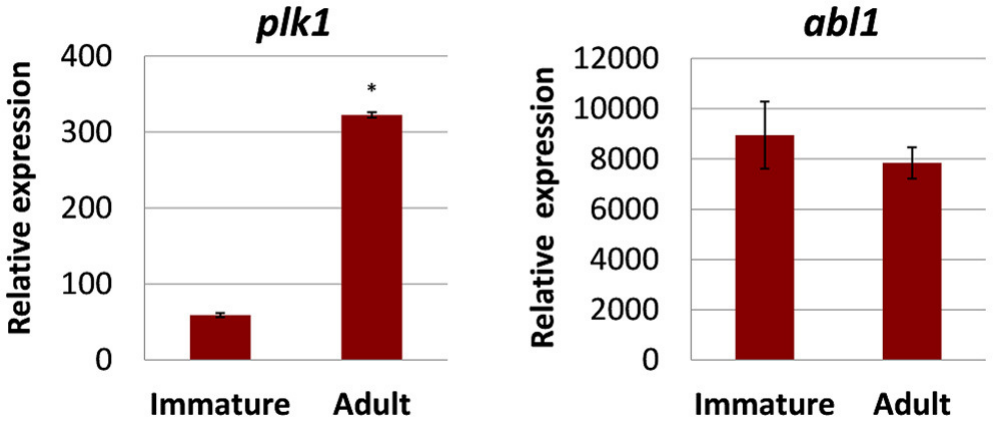
Transcriptional expression of selected kinases in different developmental stages of *Fasciola hepatica*. Relative expression in 4-week-old immature and 12-week-old adult worms was determined by qRT-PCR and normalization against two reference genes, glutamyl-prolyl-tRNA synthetase (Fh*eprs*), and tubulin-specific chaperone D (Fh*tbcd*). Data represent the mean ± SEM of three to four biological replicates. Relative expression values were calculated by the formula 2^−ΔCt^ × *f*, with *f* = 1,000 as an arbitrary factor. Significant differences are indicated with *p < 0.05 (Wilcoxon rank sum test). *abl1*, tyrosine-protein kinase ABL1; *plk1*, polo-like kinase 1. Data from (18) (modified) under the Creative Commons license (http://creativecommons.org/licenses/by/4.0/).

Before testing kinase inhibitors, we first determined the transcript (expression) levels of potentially druggable kinases in two relevant pathologic life stages of *F. hepatica*: early immature flukes (here 4 weeks age) that typically cause acute fascioliasis while migrating through liver parenchyma and the adult flukes (here 12 weeks old) residing in the bile duct during chronic fasciolosis. We focused on two kinases that were previously discussed as promising anthelminthic targets against other types of parasitic flatworms such as schistosomes (7, 8, 19, 20, 38–41), and for which we previously described putative orthologs in *F. hepatica* (18). These were the PLK1 ortholog Fh*plk1* and the ABL-TK ortholog Fh*abl1* (for accession numbers, see Supplementary Table 1). Both kinases were expressed in both developmental stages as revealed by qRT-PCR, albeit with varying levels (Figure 1). The overall transcript levels were up to 152-fold higher for Fh*abl1* compared to Fh*plk1*. Notably, Fh*plk1* was >5-fold upregulated during development to the adult stage. Taken together, Fh*plk1* and Fh*abl1* were expressed in both stages relevant for pathogenicity of fasciolosis, and Fh*plk1* exhibited a stage-dependent regulation of expression levels.

### Effect of PLK1 and ABL Kinase Inhibitors on Viability of *F. hepatica*

**Figure 2:**
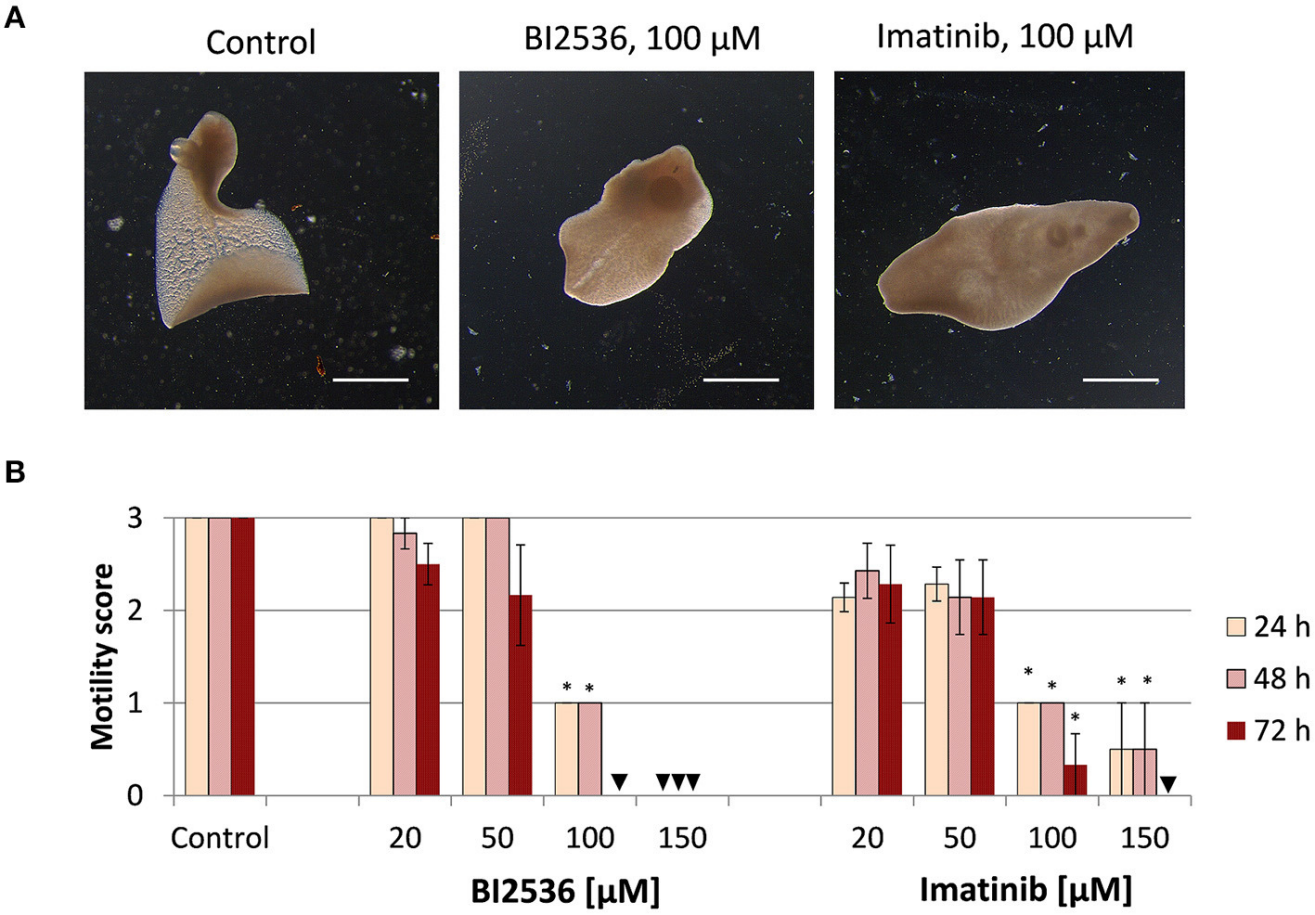
Treatment with kinase inhibitors affects motility of *Fasciola hepatica* immature flukes *in vitro*. Four-week old flukes were treated for 72 h with different concentrations (20–150 μM) of the PLK1 inhibitor BI2536 or the ABL-TK inhibitor imatinib. Motility was assessed every 24 h. Control worms were treated with the inhibitor solvent, DMSO. **(A)** Representative images taken after 72 h treatment. **(B)** Motility scores for all timepoints and concentrations (score 3 = normal, 2 = reduced, 1 = severely reduced, 0 = no motility). Data represent the mean ± SEM of two independent experiments with two to four flukes per experiment. Significant differences are indicated with *p < 0.05 (Wilcoxon rank sum test). Triangle indicates a score of zero. Scale bars correspond to 1 mm.

**Figure 3:**
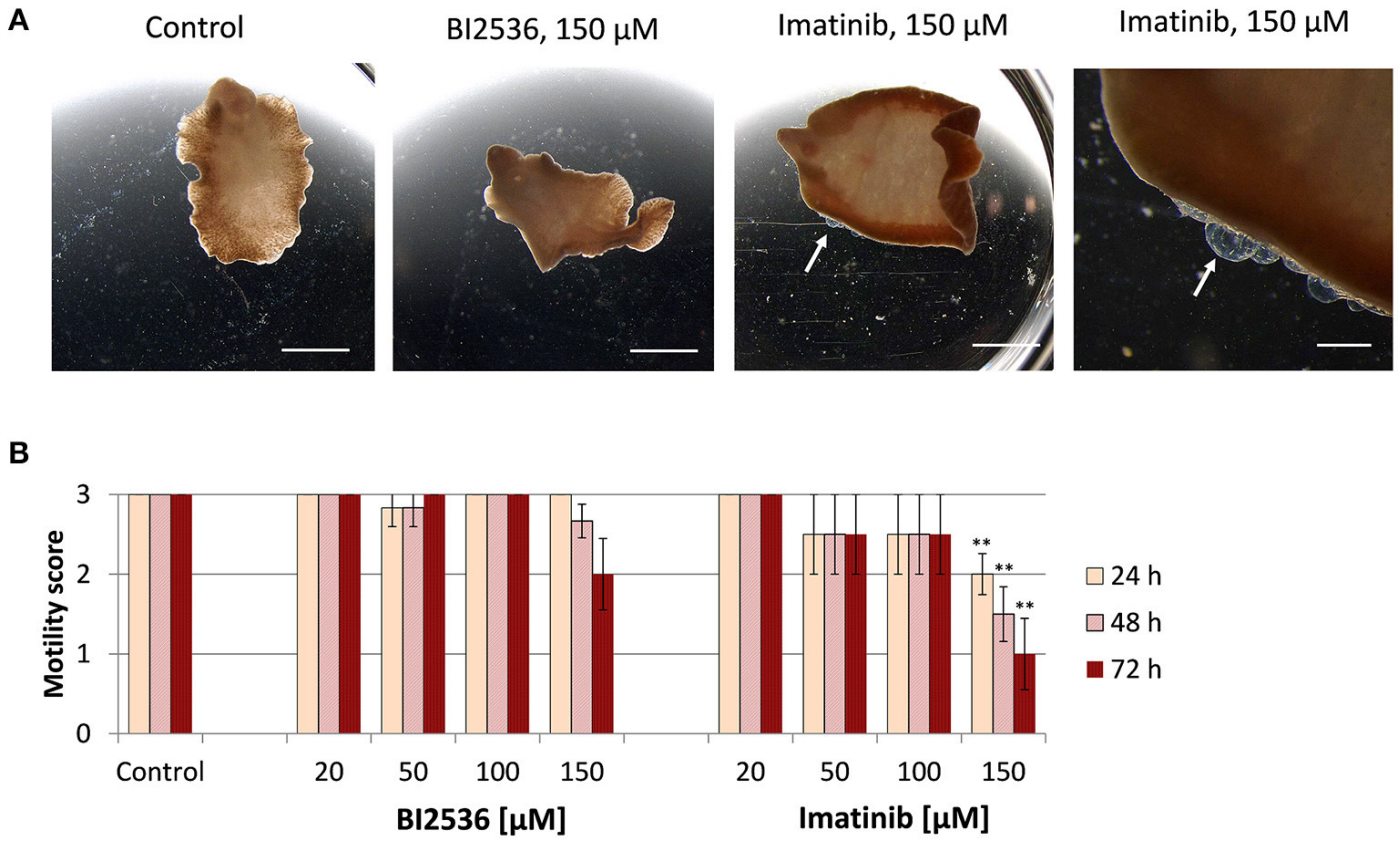
Motility of adult *Fasciola hepatica* after treatment with kinase inhibitors *in vitro*. Twelve-week-old flukes were treated for 72 h with different concentrations (20–150 μM) of the PLK1 inhibitor BI2536 or the ABL-TK inhibitor imatinib. Motility was assessed every 24 h. Control worms were treated with the inhibitor solvent, DMSO. **(A)** Representative images taken after 72 h treatment. **(B)** Motility scores for all timepoints and concentrations (score 3 = normal, 2 = reduced, 1 = severely reduced, 0 = no motility). Data represent the mean ± SEM of two independent experiments with two to four flukes per group and experiment. Significant differences are indicated with **p < 0.01 (Wilcoxon rank sum test). Scale bars correspond to 5 mm.

Next, we tested the druggability of PLK1 and ABL kinases by treating immature and adult *F. hepatica* with commercial kinase inhibitors *in vitro*. As a primary readout, fluke motility and survival were assessed every 24 h within a 72-h culture period. We tested the PLK1 inhibitor BI2536 and the ABL-TK inhibitor imatinib, at concentrations of 20–150 μM. Both inhibitors had lethal effects on immature flukes. The efficacy of imatinib (150 μM lethal after 72 h for all flukes, and 100 μM for 4 out of 6 flukes) was somewhat lower compared to BI2536 (100 μM lethal after 72 h for all flukes) (Figure 2). In adult *F. hepatica*, however, BI2536 failed to affect motility at any tested concentration. At 150 μM, imatinib was lethal for two of six adult flukes and reduced the motility of the others to a minimum (score of 1) within 72 h. In addition, some individuals had pronounced tegumental damage with bleb formation at their surface (Figure 3). Furthermore, we compared the *in vitro* efficacy of imatinib with the gold standard triclabendazole based on motility scoring of immature flukes after 72 h exposure. While imatinib clearly affected fluke vitality at 100 μM, triclabendazole reached a similar effect at 50 μM (Supplementary Figures 1A,B). Cytotoxicity of both drugs toward the murine liver cell line FL83B was comparable with EC50 values of 95.0–96.8 μM (Supplementary Figures 1C,D). Taken together, imatinib performed almost as well as triclabendazole *in vitro* and showed strong fasciolicidal effects against immature and adult flukes, while BI2536 was incapable to affect viability of adults.

### The PLK1 Inhibitor BI2536 Interrupts Egg Production

**Figure 4:**
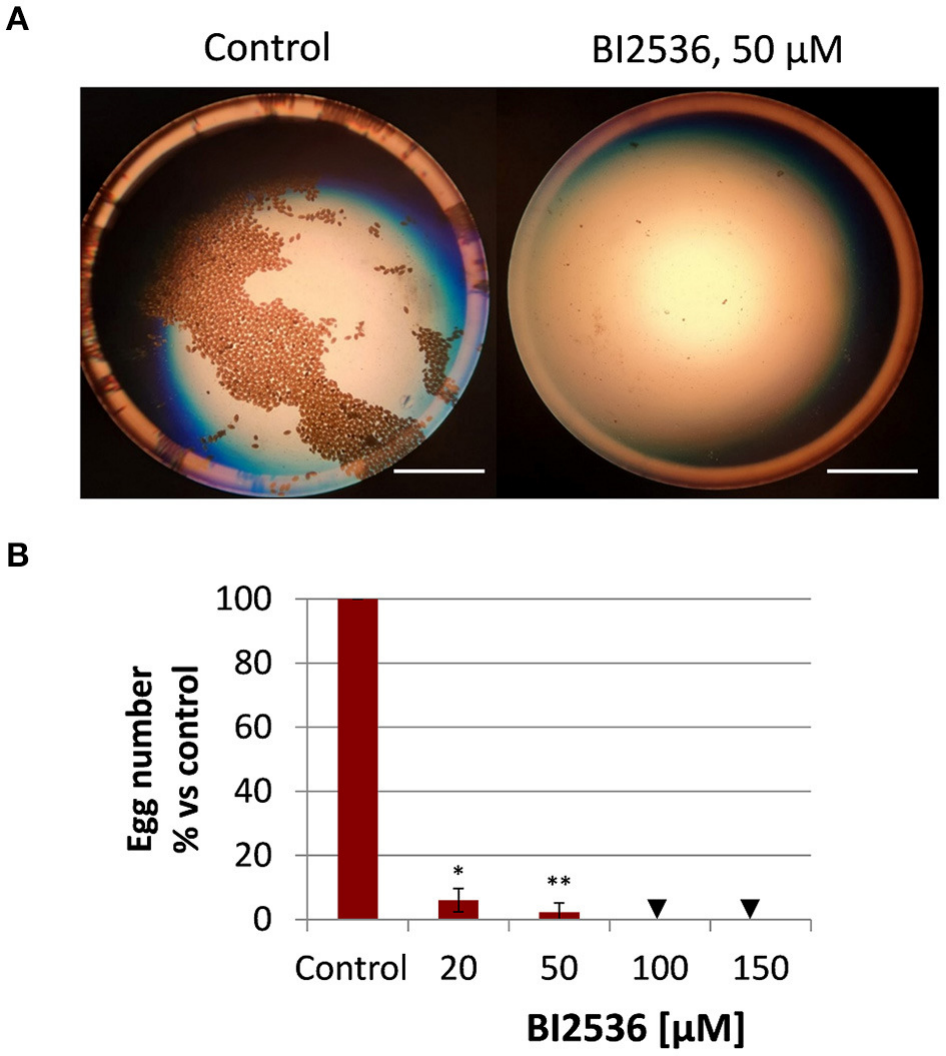
Treatment with the PLK1 inhibitor BI2536 interrupts egg production by adult *Fasciola hepatica in vitro*. Twelve-week-old flukes were treated for 72 h with different concentrations of BI2536 (20–150 μM). Control worms were treated with the inhibitor solvent, DMSO. Eggs produced in the last 24 h of the treatment period were counted. **(A)** Representative images of eggs produced by one control fluke compared to a fluke treated with 50 μM of BI2536. **(B)** Relative egg numbers compared to control flukes. Data represent the mean ± SEM of 2 independent experiments with two to four flukes per experiment. Significant differences are indicated with *p < 0.05, **p < 0.01 (Wilcoxon rank sum test). Triangle indicates a score of zero. Scale bars correspond to 5 mm.

Given the high transcript abundance of Fh*plk1* in adult worms, it was surprising to note the failure of the PLK1 inhibitor BI2536 to affect viability of the adults. Because PLK1 was found expressed in gonadal tissue of a related flatworm (22), we wondered whether BI2536 may affect reproduction. To answer this question, the number of eggs produced per adult liver fluke was quantified at the end of the 72-h treatment period and compared to the control group without treatment. A concentration as low as 20 μM significantly reduced the egg count to 6% of the amount produced by control worms, and 50–100 μM abolished egg production completely (Figure 4), while leaving motility of the worms entirely unaffected (Figure 3). Therefore, effects of PLK1 inhibition seem to be restricted to reproduction in adult *F. hepatica*, and a reproductive function of PLK1 mirrors the expression peak of this kinase at the adult but not immature developmental stage.

### Detection of the ABL Kinase Inhibitor Imatinib in Tissues of *F. hepatica* by MALDI MSI

**Figure 5:**
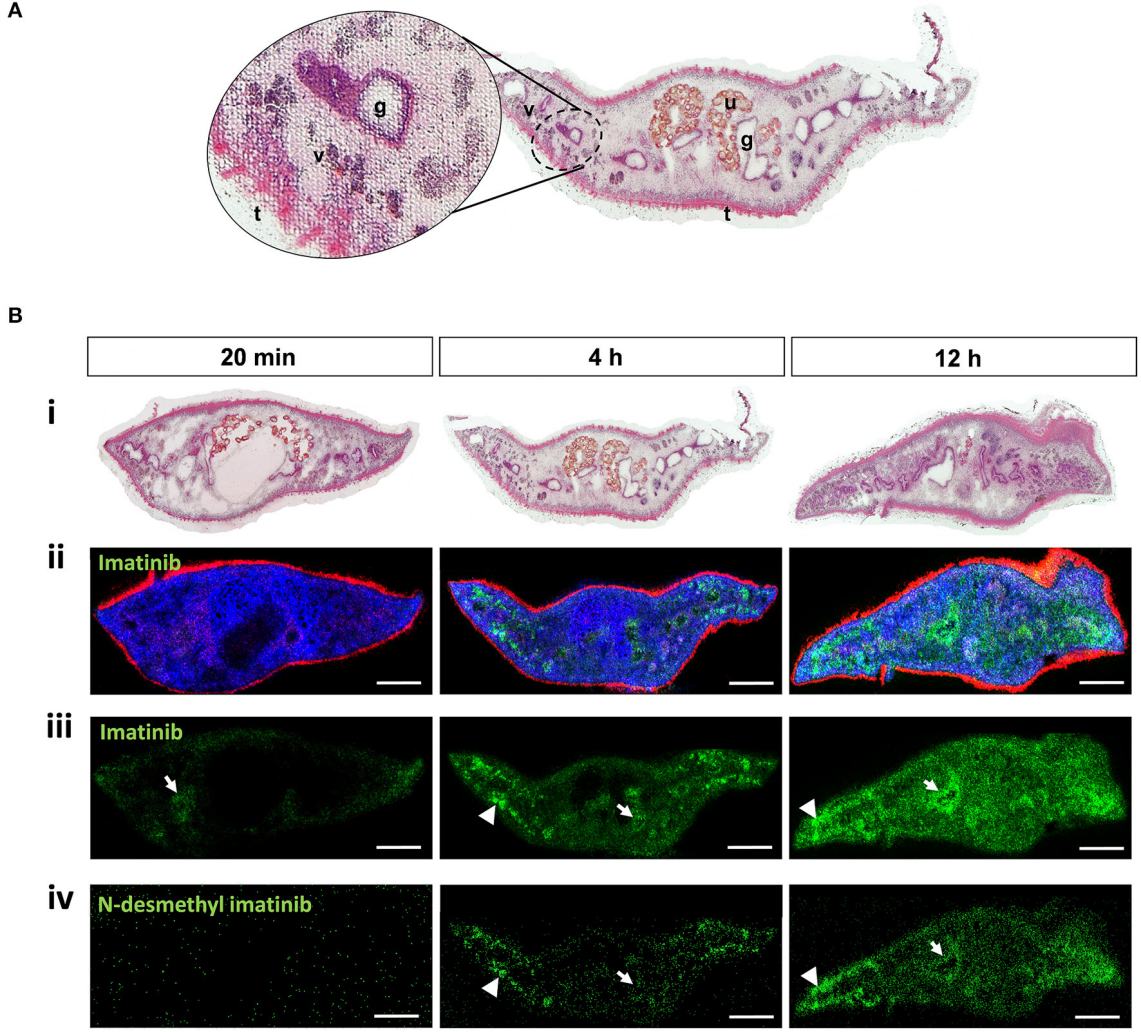
Detection of imatinib and its main metabolite in tissues of adult *Fasciola hepatica* by MALDI mass spectrometry imaging (MSI). Twelve-week-old flukes were treated for 20 min, 4 h, or 12 h with 100 μM imatinib. Compounds and endogenous lipids were detected in transversal sections using AP-SMALDI MSI, and sections were stained with H&E afterwards. **(A)** Example of an H&E-stained section showing the typical riddled pattern with numerous white spots from which tissue was laser-ablated during MALDI. Different tissues are indicated: gut/gastrodermis (g), tegument (t), uterus (u), and vitellarium (v). **(B)** For each timepoint of imatinib treatment, two flukes were analyzed, of which one representative is depicted. (i) H&E-stained sections to visualize the fluke‘s tissues after AP-SMALDI MSI measurements. (ii) RGB images from AP-SMALDI MSI showing transversal sections with the following signals: *m*/*z* 494.266464 (green, protonated imatinib), *m*/*z* 812.618589 [red, protonated PE(41:3), *m*/*z* 810.598235; blue, PC(36:1) sodium adduct]. Lipid assignment is based on LIPIDMAPS (36) results. (iii, iv) AP-SMALDI MSI single channel images showing sections depicting the imatinib signal at *m*/*z* 494.266464 corresponding to [M+H]^+^ (iii) or the metabolite N-desmethyl imatinib at *m*/*z* 480.251261 corresponding to [M+H]^+^ (iv). Arrows indicate compound localized within the intestine or gastrodermis, arrowheads within the vitellarium. Scale bars correspond to 500 μm.

We studied the tissue tropism of imatinib in adult *F. hepatica* to get a first idea of the potential uptake mode, distribution, and metabolization within tissue over time. Adult flukes were treated for 20 min, 4 h, or 12 h with 100 μM of imatinib, followed by drug imaging in transverse sections using the innovative approach of AP-SMALDI MSI, which we recently established for *S. mansoni* (42). An organic matrix was sprayed onto the liver fluke section from which a highly focused, pulsed UV laser beam ablated material in a rasterized fashion. Formed ions were then subjected to AP-SMALDI MSI analysis (43). The location of the ablated spot and its mass spectrum were matched to image the spatial distribution of analytes of a certain *m*/*z* value, such as of protonated imatinib, within the section. Finally, H&E staining of the AP-SMALDI MSI-processed sections allowed mapping of the imatinib signal to organs of the fluke. Because of the spot-wise laser ablation with a step size of 10 μm, the H&E-stained tissue displayed a riddled-like appearance after AP-SMALDI MSI. Nevertheless, tissue structures and organs such as intestinal lumen with gastrodermis, vitellarium, uterus with eggs, tegument, and subtegumental muscle layers could still be easily discriminated (Figure 5). As an additional anatomical reference in AP-SMALDI MSI images, two endogenous lipids were mapped based on LIPIDMAPS (36): phosphatidylethanolamine (PE) at *m/z* 812.618589 [protonated PE(41:3)] marking the tegument and phosphatidylcholine (PC) at *m/z* 810.598235 [PC(36:1) sodium adduct] marking the parenchyma and other inner tissues. Protonated imatinib was successfully detected in sections at *m/z* 494.266464 as can be seen in lipid/imatinib overlay images and imatinib single-channel images (Figures 5Bii–iii). Already after 20 min of incubation time, small amounts of the drug were detectable within the flukes and, as expected, an increased accumulation of imatinib was noted over time. The imatinib signal intensity in 4 and 12-h samples was approximately one order of magnitude higher than the 20-min samples (Supplementary Figure 2A). With respect to the tissue tropism, imatinib was merely found in some parts of the intestine and at surface-near tissue areas after 20 min, while the drug spread throughout the parenchyma after 4 h, with the highest signal intensities in vitellarium tissue. The uterus and intestinal lumina remained negative, while intestinal walls (presumably the gastrodermis) were positive for imatinib. After 12 h, imatinib signal intensities further increased and spread throughout the body, again with the highest accumulation in intestinal walls and vitellarium (Figure 5B).

In humans, imatinib is metabolized and forms N-desmethyl imatinib as the main bioactive metabolite, possibly contributing to drug activity. We therefore wondered whether a similar metabolization takes place in *F. hepatica*, and how fast the metabolite signal would occur. Indeed, protonated N-desmethyl imatinib was detected in sections of the imatinib-treated flukes at *m/z* 480.251261 (Figure 5Biv). While imatinib was detected already after 20 min of incubation, the metabolite occurred time-delayed after 4 h, and further accumulated after 12 h. The metabolite's tissue tropism was similar to that of imatinib, with the highest signal intensities in the vitellarium. For the timepoints where the metabolite was detectable, its average signal intensity was 1.5–2 orders of magnitude lower than the average imatinib signal intensities (Supplementary Figure 2B). While average signal intensities of imatinib and the metabolite increased over time, signal intensities of the lipid PC (36:1), here used as control signal, largely remained stable. A representative mass spectrum of a sample from 12-h imatinib exposure depicts the signals of imatinib and the metabolite as well as corresponding intensities (Supplementary Figure 3). The spectrum belongs to one single pixel that is located in the vitellarium region on the right side of the worm section (Figure 5B at 12 h), where the drug and the metabolite accumulated. Root-mean-square error (RMSE) plots of the signals at *m*/*z* 494.266464 (assigned to protonated imatinib) and *m*/*z* 480.251261 (assigned to protonated N-desmethyl imatinib) showed one single peak with no shoulders within an *m*/*z* bin of 5 ppm width (Supplementary Figure 2), which indicates that only the analyte was detected and every green pixel in the MALDI images actually represents imatinib and its metabolite, respectively. N-demethylation of imatinib to N-desmethyl imatinib is mediated via the liver cytochrome P450 enzymes CYP2C8, CYP3A4, CYP3A5, and CYP3A7 in humans (44, 45). DeltaBLAST searches predicted a putative ortholog for CYP2C8 in *F. hepatica*. SMART protein domain analysis revealed an N-terminal transmembrane domain (Supplementary Figure 4) that is typical for this type of microsomal cytochromes, which form integral membrane proteins located in the endoplasmic reticulum (46).

**Figure 6:**
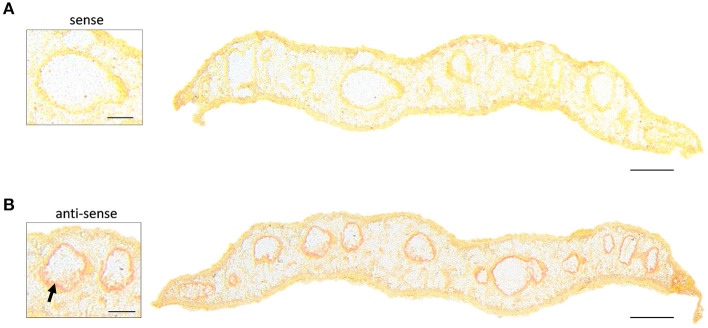
*In situ* hybridization localizes transcripts of Fh*abl1* kinase in gastrodermal tissue of adult *Fasciola hepatica*. ISH was performed on transverse sections of 12-week-old *F. hepatica* using digoxygenin-labeled sense probes for Fh*abl1* as negative control **(A)**, or anti-sense probes to detect Fh*abl1* transcripts **(B)**. Positive, red staining localized to the gastrodermis (arrow). Scale bars correspond to 250 μm (whole sections) or 100 μm (inserts).

Taken together, AP-SMALDI MSI-assisted drug imaging of imatinib in adult *F. hepatica* revealed (a) a fast incorporation of the drug within 20 min, (b) an accumulation over time especially in intestinal and vitellarium tissue, and (c) a metabolization to the same bioactive metabolite as found in humans. To investigate whether drug distribution correlated with target distribution, we performed *in situ* hybridization to localize Fh*abl1* transcripts in tissue sections of adult *F. hepatica*. A moderate positive staining was found for the gastrodermis (Figure 6). Thus, the localization of both imatinib as drug and its target largely matched each other.

### Gene Expression Changes in Adult Flukes After Imatinib Treatment

**Figure 7:**
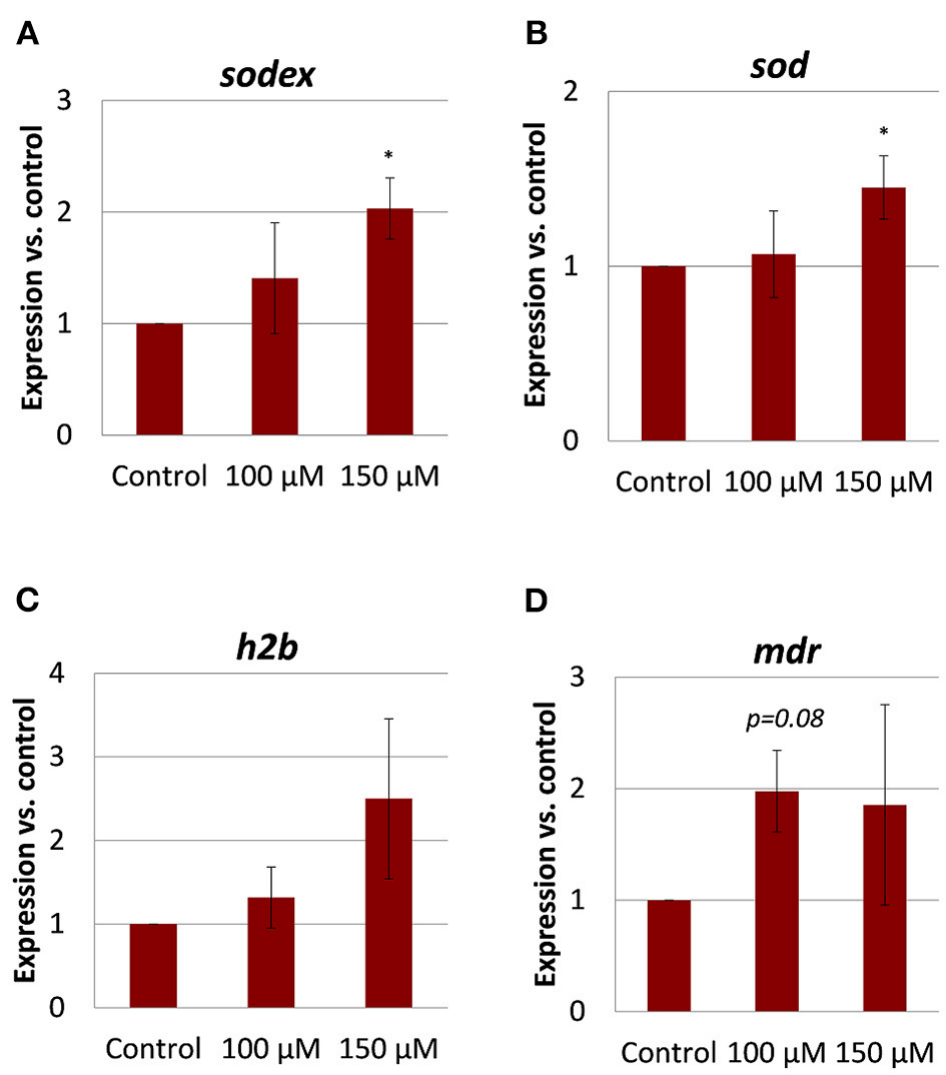
Gene expression changes in adult *Fasciola hepatica* after imatinib treatment. Twelve-week-old flukes were treated for 72 h with 100 or 150 μM imatinib *in vitro*, and the fold change of transcripts of selected genes relative to DMSO-treated worms was quantified by qRT-PCR. Normalization was done against two reference genes, glutamyl-prolyl-tRNA synthetase (Fh*eprs*) and tubulin-specific chaperone D (Fh*tbcd*). Of interest were **(A)** extracellular superoxide dismutase (*sodex*) and **(B)** superoxide dismutase (*sod*) as genes involved in the defense toward oxidative stress, **(C)** the histone *h2b* important for DNA duplication during the cell cycle, and **(D)** a putative multidrug resistance gene (*mdr*). Data represent the mean ± SEM of two independent experiments with three to five flukes per group. Significant differences vs. control are indicated with *p < 0.05 (Wilcoxon rank sum test).

A previous microarray study revealed substantial transcriptional changes in adult *S. mansoni* upon imatinib treatment, which suggested a wide influence of the inhibitor on worm physiology (24). For imatinib-treated *F. hepatica*, we expected a similar impact on the expression of physiologically important genes. To test this, we quantified the expression of a collection of genes selected from the *S. mansoni* study after identifying potential orthologs in *F. hepatica* (for gene ID numbers, see Supplementary Table 1): the multidrug resistance protein 1 (MDR1, also called ABCB1 or P-glycoprotein) belonging to the group of drug efflux pumps; histone 2B (H2B), which is enriched during cell cycle (47); two types of Cu/Zn superoxide dismutases (SOD), i.e., a predicted extracellular SOD (SODex) and a cytosolic SOD, both first-line defense proteins against oxidative stress (48). Orthologs were identified by BLASTp search of known genes in *S. mansoni* against the genome of *F. hepatica*, and correct annotation of the hits was further confirmed by another BLASTp search against *Homo sapiens* (accession numbers see Supplementary Table 1). SMART analysis confirmed the presence of conserved protein domains (Supplementary Figure 5A). The gene products of Fh*h2b*, Fh*sodex*, and Fh*sod* were previously identified by proteomic analyses (49–51), and their annotation was confirmed in our analysis. It was previously shown for a related flatworm species that oxidative stress upregulated the expression of SOD on a transcriptional level (52). The highest amino acid sequence identity to human MDR1 was found for the product of a gene, which we called Fh*mdr*. This is different from the protein that was named “MDR1” in a previous study of *F. hepatica* (53), but which turned out to have a lower amino acid sequence identity to human MDR1 and a lower e-value in BLASTp results compared to the here described Fh*mdr* gene product (Supplementary Figure 6). A hidden Markov model predicted the correct topology of the newly described membrane protein FhMDR. It contained the typical two transmembrane domains that each consist of several transmembrane helices and are interlinked by a large cytoplasmic domain with an ATP-binding site (Supplementary Figure 5B). qRT-PCR analyses revealed trends of upregulation for all four marker genes in adult flukes treated with imatinib when compared to control flukes (Figure 7). In detail, Fh*sod* and Fh*sodex* expression were significantly increased upon treatment with 150 μM imatinib, and the same trend was found for Fh*mdr* in flukes treated with 100 μM. Fh*h2b* transcript levels were 2–4-fold elevated in two out of three imatinib-treated worms (150 μM). Because the increase for Fh*h2b* and Fh*mdr* transcript levels was not significant, a drug-induced effect on these genes remains uncertain. Taken together, imatinib modified the expression of some genes with expected physiological importance not only in *S. mansoni*, but also in *F. hepatica*.

## Discussion

Protein kinases have been propagated as valuable drug targets against various pathogens, from bacteria to protozoan and helminth parasites (54–56). In liver flukes, however, until now only little attention was paid to kinases and kinase inhibitors. In our study, (1) we demonstrated the *in vitro* anthelminthic activity of two different kinase inhibitors against different pathogenic, intramammalian stages *F. hepatica*, (2) we demonstrated the distribution of the ABL kinase inhibitor imatinib and its presumed target within fluke tissue, and (3) we highlighted possible modes of action of imatinib by expression analysis of selected genes with suggested importance for fluke biology.

### Kinases as Therapeutic Target in *F. hepatica*

An ideal drug target should be expressed and physiologically relevant in all pathogenic life stages of *F. hepatica*: in immature flukes migrating and feeding within the liver parenchyma, as well as in adult flukes that feed in the bile duct and cause chronic infections (5). This ensures that its pharmacological inhibition can have cross-stage activity. In line with this, the ABL1 and PLK1 kinases addressed in this study were transcriptionally expressed in immature as well as adult flukes. These highly conserved kinases fulfill important cellular functions as known from humans and model organisms, and we hypothesize that the same holds true for liver flukes. PLK1 proteins are key regulators of the cell cycle progression during M-phase (10). ABL-TK regulate a variety of cellular processes such as cytoskeletal rearrangement and cell differentiation (11). The highly conserved nature of the selected kinases makes inhibitory activities in exotic species such as liver flukes likely.

To target members of these PK families, we focused on two well-described anti-cancer drugs with proven antischistosomal activity. Imatinib, also known as Gleevec or Glivec (Novartis, Basel, Switzerland; formerly referred to as STI571 or CGP57148B), inhibits protein TKs of the ABL-family and is used for therapy of chronic myeloid leukemia in humans (57). BI2536 is a small-molecule inhibitor of PLK1, which belongs to the group of serine/threonine-protein kinases. The ideal fasciolicidal compound should target all relevant life stages within the host. Currently, mainly the gold standard drug triclabendazole is cross-stage active, while other drugs in use mainly target the adult stage (6). Among the tested kinase inhibitors, imatinib fulfilled this criterion of cross-stage activity. Imatinib was lethal to immature and adult *F. hepatica* at 100–150 μM. This represents a two to three times higher active concentration compared to the current gold standard triclabendazole and two other common drugs in use, clorsulon and closantel, for which we established lethal concentrations of 50 μM in our *in vitro* culture system. Opposite to imatinib, the PLK1 inhibitor BI2536 had basically no effect on adult liver fluke viability, but killed immature liver flukes. It appears that BI2536 in general has stronger effects on the juvenile stages of flukes compared to their adult stages. Also for the blood fluke *S. mansoni*, juvenile stages died upon inhibitor treatment or interference with PLK1 expression using RNAi, while the viability of adult worms was unaffected (20, 22). We hypothesize that the described role of PLK1 in mitosis control might be of particular importance during the growth phase of juvenile flukes, when a high proliferative activity of somatic stem cells (neoblasts) might be needed. In the adult stage, however, PLK1 function may be more crucial to ensure egg production, i.e., germline stem cell proliferation, which is certainly an important function for the parasite but not essential for its survival. The critical role for reproduction of *Fasciola* was also underlined by the drop of egg release upon PLK1 inhibitor treatment. Also in *S. mansoni*, the mitotic function of PLK1 in germinal cells was important for normal egg production (22). Thus, although BI2536 is the most potent and selective PLK1 inhibitor available (58) and was shown to inhibit the catalytic activity of recombinant *S. mansoni* PLK1 even at nanomolar doses (22), targeting PLK1 might not be the most promising anthelminthic strategy. Because of its activity against early immature liver flukes, it might still be interesting as a combination therapy with other drugs for which the activity spectrum is restricted to older stages, such as clorsulon and closantel (6).

### Drug Imaging as Powerful Tool for Compound Studies in *F. hepatica*

To address the kinetics of drug uptake and tissue tropism in *F. hepatica*, we applied for the first time MALDI MSI in this parasite. We managed to visualize an increasing accumulation of imatinib in different tissues from 20 min to 12 h of exposure, and could prove that imatinib is metabolized by *F. hepatica* the same way as in humans. The strong signal intensities in tissues lining the intestinal tract, probably gastrodermis, suggest an oral uptake of imatinib. A second, additional uptake route may occur via the tegument, the physiologically active outer surface layer of flukes (59). Although the tegument was not found positive for imatinib in AP-SMALDI MSI, a tegumental uptake cannot be fully excluded. Such a tegumental uptake of the drug clorsulon has been indirectly demonstrated before by ligature of the anterior body part to prevent oral ingestion (60). The pronounced tegumental damage observed in *F. hepatica* further argues for a tegumental targeting by imatinib. That the metabolite's signal intensities were about two orders of magnitude lower than the imatinib signal intensities suggests either an incomplete metabolization of imatinib or, alternatively, a slow metabolization of continuously incorporated imatinib. As in humans (44), cytochrome p450 2C8 might catalyze the metabolization of imatinib. N-desmethyl imatinib is pharmacologically active and shows a similar potency as the parent drug including potent ABL inhibition in the nanomolar range (45). It was shown to inhibit cell proliferation and to induce apoptosis in human cells (45, 61). Thus, the metabolite likely contributes to the fasciolicidal effect seen after imatinib treatment.

Our lab has previously established and optimized AP-SMALDI MSI for the visualization of lipid distributions in *S. mansoni* (42). Recently, we also established drug imaging for this species, utilizing imatinib as proof of principle (Mokosch et al., under review). Our data suggest that *S. mansoni* and *F. hepatica* have different uptake efficiencies for imatinib when applied at the same concentration of 100 μM. The drug was distributed throughout internal tissues of adult males of *S. mansoni* already after 20 min of exposure (Mokosch et al., under review), while only traces of imatinib were detected within the same time in *F. hepatica*, and it took 1 h for a clear drug accumulation. In case of a tegumental drug uptake, the considerably thicker tegument of *F. hepatica* with around 20 μm (62) compared to *S. mansoni* with around 4 μm (63) might contribute to this delayed drug internalization. In case of an oral drug uptake, the larger body size and higher degree of intestinal branching in adult liver flukes compared to blood flukes might delay drug distribution within internal tissues. Nevertheless, within 12 h, imatinib was found distributed throughout the body of *F. hepatica*.

Taken together, AP-SMALDI MSI revealed a distinct uptake kinetic for imatinib, and it demonstrated the accumulation of imatinib in specific parasite tissues. In one of these tissues, highest expression of the putative kinase target was found. Furthermore, the obtained data support the hypothesis that *F. hepatica* incorporates imatinib orally and metabolizes it to a known bioactive metabolite.

### Imatinib's Possible Mode of Action in *F. hepatica*

To shed some first light on a possible mode of action, we studied the transcriptional expression of various genes thought to be important for homeostasis. We focused on representative genes involved in the regulation of oxidative stress, cell cycle, and drug efflux. The selection of genes was inspired by a microarray study in *S. mansoni*, which revealed substantial transcriptional changes caused by imatinib treatment. Overall expression changes in gut-, muscle-, tegument- and gonad-associated genes pointed to a broad negative effect of imatinib on schistosome physiology (24). In imatinib-treated *F. hepatica*, the Fh*mdr* drug resistance gene, encoding for a drug efflux pump, was transcriptionally upregulated, which might be one mode how the fluke tries to defend against toxic compounds such as imatinib. Indeed, MDR1 has been predicted to be a drug or chemical exporter in helminths (64). While the transcriptional level of two *mdr* genes in *S. mansoni* was downregulated in response to imatinib (24), it was upregulated by praziquantel treatment (65). Eventually, *mdr* expression and its upregulation was obviously ineffective in *F. hepatica*, as imatinib was still able to affect fluke viability. It might nevertheless be interesting to treat flukes with imatinib under pharmacological inhibition of MDRs to test for an even higher drug efficacy.

*Sod* genes were transcriptionally upregulated by imatinib in both fluke species, which might reflect an increased oxidative stress response (52). Reactive oxygen species cause oxidative DNA damage (66), and we speculate that this might contribute to the anthelminthic effect of imatinib. TK inhibitors such as imatinib are known to cause myotoxicity as a side effect, which was associated with mitochondrial superoxide accumulation and increased SOD expression (67). Thus, imatinib might act as mitochondrial toxicant also in *F. hepatica* by inducing reactive oxygen species and an increase of SOD expression as part of the anti-oxidant defense system. In this context, it is important to note that oxidative stress and chemical inhibition of ABL-TK activity arrest cells at the S phase of the cell cycle (68–70). We found a trend of increased *h2b* expression after imatinib treatment of worms. *H2b* transcriptional expression increases to high levels when cells enter the S phase to provide histones for packing the newly synthesized DNA (71). We therefore speculate that increased *h2b* transcript levels might reflect an S-phase arrest in imatinib-treated *F. hepatica*, which may be caused by drug-induced oxidative stress. In conclusion, imatinib's anthelminthic mode of action might involve the induction of oxidative stress and/or interference with cell cycle progression. Future studies involving cell cycle analyses and quantification of ROS should prove whether this hypothesis holds true.

### Outlook

Our study demonstrates that kinases are worth to be considered as therapeutic targets in *F. hepatica*. The finding that the ABL-TK inhibitor imatinib displays cross-species activity against schistosomes (19) but also *F. hepatica* as a pathogen of tremendous veterinary importance makes this drug even more attractive for further development. Importantly, imatinib displayed cross-stage activity against liver flukes, a benchmark set by the current gold standard triclabendazole. These results motivate for further validation in animal studies. In case of imatinib, serum proteins such as alpha-1-acid glycoprotein (AGP) were shown to bind the drug, which reduced its *in vivo* efficacy against schistosomes using rodents as model host (72). More motivating was a case report on orally administered imatinib in a human filariasis patient, which reduced worm burden (73). Thus, when it comes to *in vivo* studies, it needs to be considered that the degree of drug-plasma protein binding varies between species, especially between rodents and human (72). AGP concentrations vary between healthy to pathological states, and AGP exhibits species-dependent binding affinities (74–76). Various drugs were found to bind with lower affinity to AGP from sheep compared to AGP from several other species (77, 78), which makes sheep as a natural host of *F. hepatica* likely suitable for imatinib trials. Next to testing the *in vivo* efficacy of imatinib against liver flukes, another important task will be the biochemical and genetic target validation for *abl* kinases in *F. hepatica*. Finally, in-depth analyses of the kinome should be undertaken, which recently predicted the expression of 455 protein kinases in the related fluke *F. gigantica* (79). The availability of kinome data for *F. hepatica* will certainly boost the discovery of potentially druggable kinases in this parasite.

## Data Availability Statement

The original contributions presented in the study are included in the article/Supplementary Materials, further inquiries can be directed to the corresponding author/s.

## Ethics Statement

The animal study was reviewed and approved by Regional Council (Regierungspraesidium) Giessen (approval number V54-19c20 15 h 02 GI 18/10 Nr. A16/2018).

## Author Contributions

SH: conceptualization. CM, HH, OP, LH, MR, ER, DD, AM, BS, and SH: methodology. CM, HH, OP, MR, LH, and SH: investigation. ER, BS, and SH: resources. CM and SH: writing—original draft preparation. CM, MR, and SH: visualization. AM, DD, ER, MR, BS, and SH: supervision. BS and SH: funding acquisition. All authors: writing—review and editing.

## Conflict of Interest

BS is a consultant of TransMIT GmbH Giessen. The remaining authors declare that the research was conducted in the absence of any commercial or financial relationships that could be construed as a potential conflict of interest.
