# Supplementary material for: Targeting Kinases in Fasciola hepatica: Anthelminthic Effects and Tissue Distribution of Selected Kinase Inhibitors
Source: Front Vet Sci. 2020 Dec 21;7:611270. doi: 10.3389/fvets.2020.611270 (PMC7779637; doi:10.3389/fvets.2020.611270)
Supplement: Supplementary file 3 [file Data_Sheet_1.pdf]

Supplementary Material

1 Supplementary Tables

Supplementary Table S1: Gene ID numbers of potential orthologues found in *Fasciola hepatica*, orthologues in *Schistosoma mansoni* and *Homo sapiens*, and known function of proteins.

**Gene of interest**	**Gene ID in *F. hepatica***	**Homology (e-value) vs. *S. mansoni****	**Homology (e-value) vs. *H. sapiens*****	**Protein function**
Fh*abl1*	maker-scaffold10x_1995_pilon-snap-gene-0.46	Abl kinase 1, Smp_246700.1 (0.0)	ABL proto-oncogene 1, non-receptor tyrosine kinase, NP_005148.2 (8e-132)	Protein tyrosine kinase involved in a variety of cellular processes, including cell division, adhesion, differentiation, and response to stress.
Fh*plk1*	maker-scaffold10x_784_pilon-snap-gene-0.36	Serine/threonine-protein kinase PLK1, Smp_009600 (1.1e-164)	Serine/threonine-protein kinase PLK1, NP_005021.2 (0.0)	The serine/threonine protein kinase is highly expressed during mitosis and performs several important functions throughout the M phase of the cell cycle.
Fh*h2b*	augustus_masked-scaffold10x_2264_pilon-processed-gene-0.1	Histone H2B, Smp_036220.1 (1.5e-51)	Histone H2B type 2-E, NP_003519.1 (3e-64)	Histones are nuclear proteins which form the basic structure of chromatin. This protein has a replication-dependent activity.
Fh*sodex*	marker-scaffold10x_713_pilon-snap-gene-0.105	Extracellular superoxide dismutase [Cu-Zn], Smp_174810.1 (3.8e-42)	Extracellular superoxide dismutase [Cu-Zn] preproprotein, NP_003093.2 (2e-27)	SODs are antioxidant enzymes that catalyze the conversion of superoxide radicals into hydrogen peroxide and oxygen which protect tissues from oxidative stress.
Fh*sod*	marker-scaffold10x_61_pilon-snap-gene-0.36	Superoxide dismutase [Cu-Zn], Smp_176200.1 (5.3e-70)	Superoxide dismutase, NP_000445.1 [Cu-Zn] (1e-60)	The encoded isozyme is a soluble cytoplasmic protein which acts to convert superoxide radicals to molecular oxygen and hydrogen peroxide.
Fh*mdr*	marker-scaffold10x_1211_pilon-augustus-gene-0.53	Smdr2, Smp_055780.1 (2.2e-132)	ATP-dependent translocase ABCB1 isoform 2, NP_000918.2 (0.0)	This protein is a member of the MDR/TAP subfamily which is involved in multidrug resistance.

* Determined by BLASTp using WormBase ParaSite, version WBPS13 (https://parasite.wormbase.org)

**Determined by NCBI BLAST (https://blast.ncbi.nlm.nih.gov/)

Supplementary Table S2: Primer sequences for quantification of transcript levels in adult *Fasciola hepatica* by qRT-PCR, and PCR product sizes.

**Gene**	**Annotation**	**Forward primer (5‘-3‘)**	**Reverse primer (5‘-3‘)**	**PCR product size (bp)**
tbcd	Tubulin-specific chaperone D	CAGCAGCCGCATTTCAGGA	AGCCAAATGGTCAATC ATCGC	176
eprs	Glutamyl-prolyl-tRNA synthetase	TACACCACAACCATCGAG GC	GTGGTCAATCCCCACGA GTT	167
abl1	ABL proto-oncogene 1,non-receptor tyrosine kinase	CTGCCTGTACATACTGTGC C	GTGCGGTGAGTTCATGG TTC	154
plk1	Polo-like kinase 1	ATGGCTAGTAAGGACGCT GC	TGCTTTCGGAACCACTT TTCC	177
h2b	Histone H2B	GTGCTGCGCCAAGTTCATC	TCTGCTCGTGATCGTTG ACC	147
sodex	Extracellular superoxide dismutase	CAGATACCGCATCCCGTCA T	TCTTGACCTGCGTGTAC GAC	151
sod	Superoxide dismutase	TTGGCGATCTGGGAAATGT TG	GACCGAGGTCGTCCTCA TT	141
mdr	ATP-dependent translocase ABCB1	CAGAAGGTTACAAAACAG TACTAG	TCGATGTGCCACTACGA CCA	215

2 Supplementary Figures

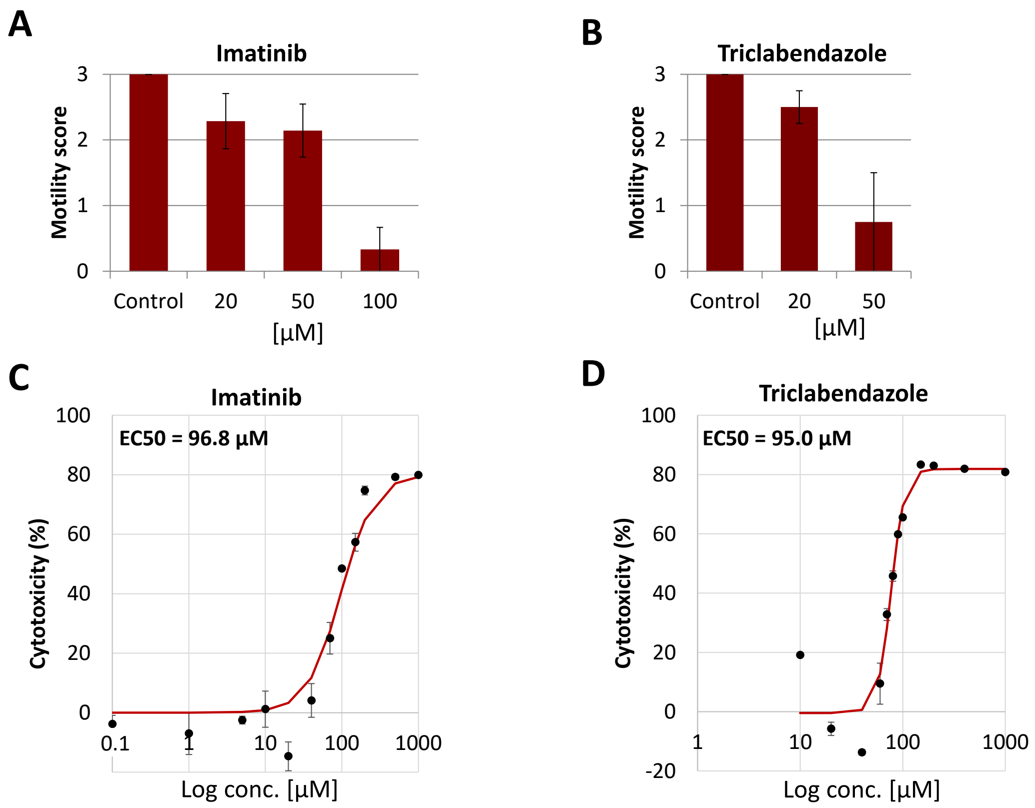

Supplementary Figure S1 Efficacy and cytotoxicity of imatinib compared to triclabendazole *in vitro*. (A, B) Four-week old *F. hepatica* flukes were treated for 72 h with different concentrations (20−100 µM) of imatinib (A) or triclabendazole (B), or were treated with the inhibitor solvent, DMSO (control). Data represent the mean±SEM of 2 independent experiments with 4 flukes per group and experiment. **(C, D)** Cytotoxicity of different concentrations (0.1−1000 µM) of imatinib (C) or triclabendazole (D) against the murine liver cell line FL83B was determined by MTT assay. Nonlinear least-squares data fitting was performed on values from two independent experiments and EC50 values were calculated. The mean of two independent experiments ± SEM is depicted.

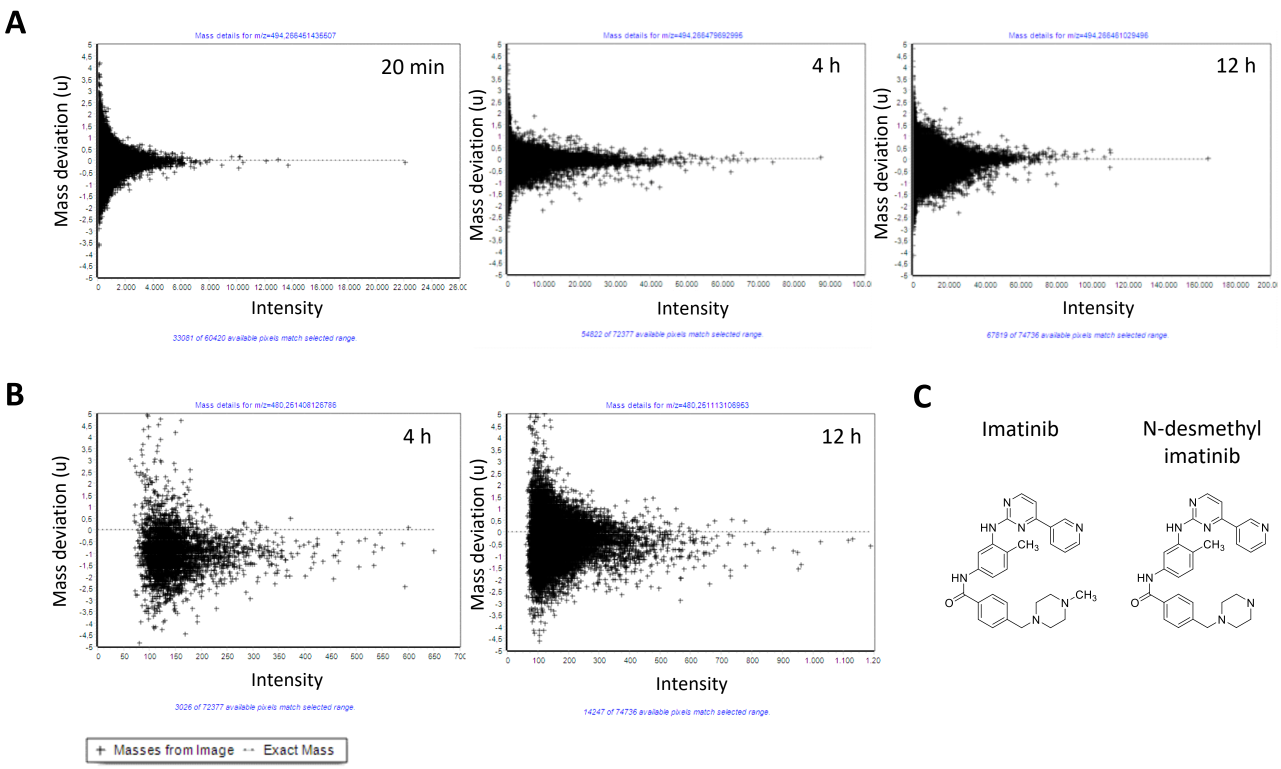

Supplementary Figure S2 Root-mean-square-error (RMSE) plots of the protonated imatinib signal at *m/z* 494.2665 (A) and the protonated imatinib metabolite signal (N-desmethyl imatinib) at *m/z* 480.2513 (B). The gaussian shaped single-peak signal gives evidence to the mass assignment. From left to right, the plots belong to the representative sections depicted in Figure 5 for 20 min, 4 h and 12 h (A) or 4 h and 12 h (B) incubation of adult *Fasciola hepatica* with imatinib (100 µM). **(C)** Chemical structures of imatinib and the metabolite N-desmethyl imatinib.

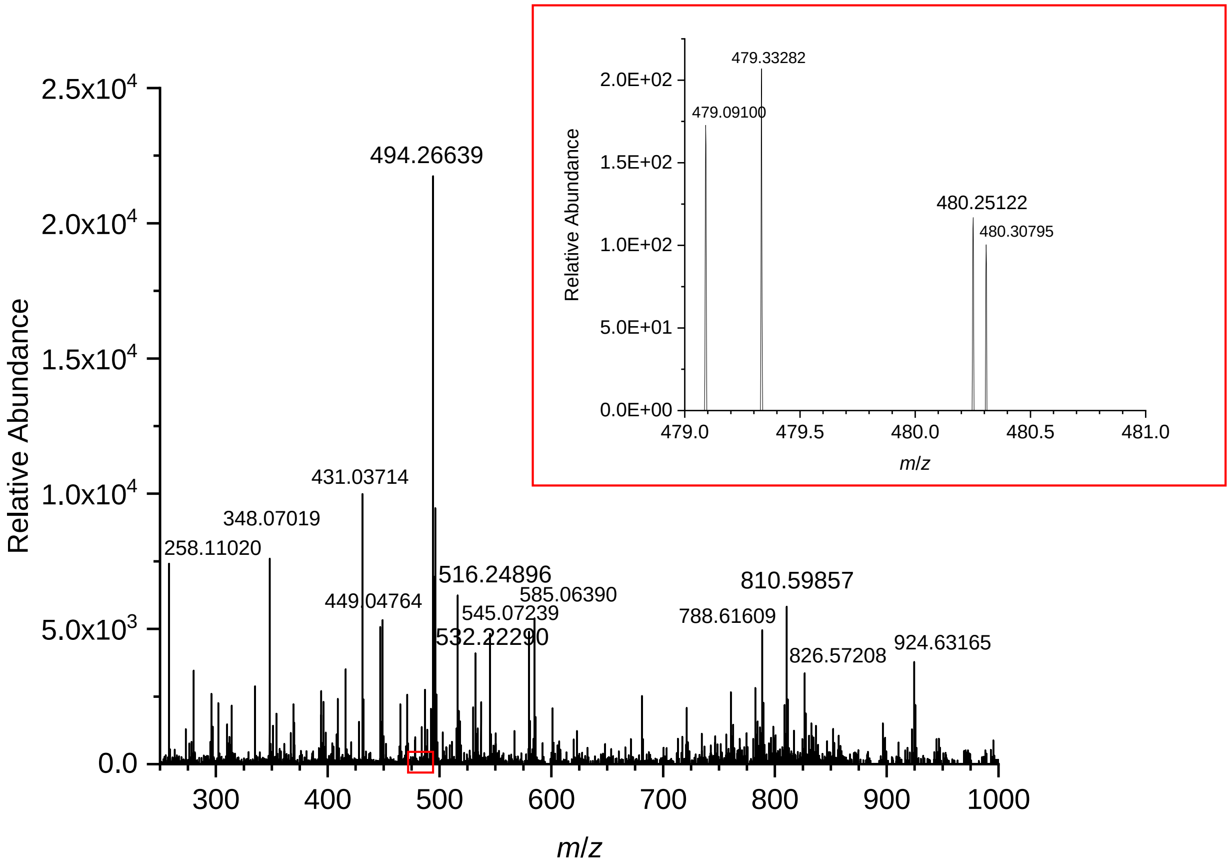

Supplementary Figure S3 One-pixel mass spectrum from AP-SMALDI MSI measurement of a *Fasciola hepatica* section. A tissue section of a worm treated for 12 h with 100 µM imatinib was processed with AP-SMALDI MSI. The mass spectrum of one representative pixel is shown. The pixel is located in the vitellarium region on the right side of the worm section depicted in Figure 5B (12 h). The spectrum shows signals at *m/z* 494.26639 (imatinib, $[M+H]^+$) and *m/z* 480.25122 (N-desmethyl imatinib, $[M+H]^+$) as well as the sodium and potassium adduct signals of imatinib at *m/z* 516.24896 and *m/z* 532.22290, and the lipid PC (36:1) at *m/z* 810.25122. Some other highly intense signals are also superscribed. The insert shows a close-up of the *m/z* range 479.0–481.0.

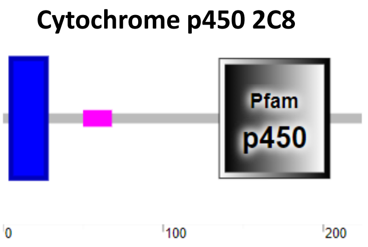

Supplementary Figure S4 Identification of conserved protein domains using SMART analysis predicts an orthologue for cytochrome p450 2C8 in *Fasciola hepatica*. In blue: transmembrane domain. Accession number: maker-scaffold10x_135_pilon-augustus-gene-0.44. The amino acid sequence was extracted from the public domain tool WormBase ParaSite.

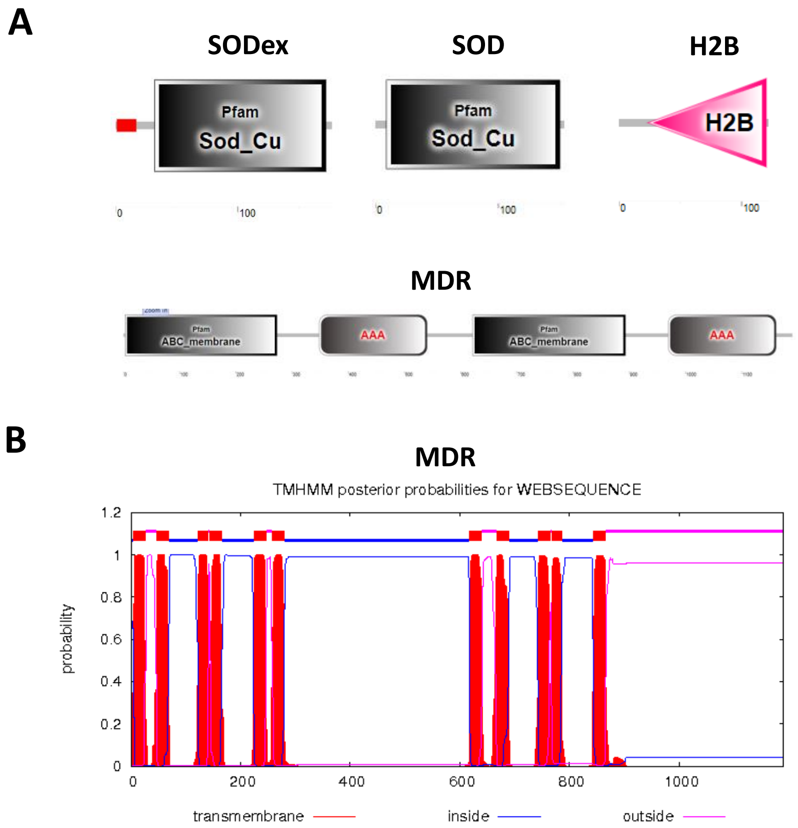

Supplementary Figure S5 Confirmation of conserved protein domains using SMART analysis (A) and of membrane protein topology using TMHMM (B) in orthologous genes predicted for *Fasciola hepatica*. Amino acid sequences were extracted from the public domain tool WormBase ParaSite. Gene accession numbers are listed in Supplementary Table S1. SODex, extracellular superoxide dismutase; SOD, superoxide dismutase; h2b, histone h2b; MDR, multidrug resistance protein. AAA, ATPase domain. The red mark in the SODex graphic represents the typical signal peptide for secretion of extracellular proteins. MDR contains the typical two transmembrane domains (ABC_membrane) which each consist of several transmembrane helices and are interlinked by a large cytoplasmic domain with an ATP-binding site (AAA).

A

Multiple sequence alignment of multidrug resistance proteins

```
HsMDR1        MDLEGDRNGGAKKKNFFKLNNKSEKDKKEKKPTVSVFSMFRYSNWLDKLYMVVGTLAAII 60
MmMDR1        MEFEENL-KGRADKNFSKMGKKSKKEKKEKKPAVGVFGMFRYADWLDKLCMILGTLAAII 59
FhMDR         ---------------------------------------------MCIVFGFIFSVA 12
FhMDR1        -------------MEKQQTENVAKSEKKKEKRNISFHKLFKYATTGEKITLVIGFIFAIA 47
FhMDR_179     --------------------------------------FSHADARDKACIFFGSIFAIA 21
                                                        :..* : ::

HsMDR1        HGAGLPLMMLVFGEMTDIFANAGNLEDLMSNITNRSDINDTGFF--MNLEEDMTRYAYYY 118
MmMDR1        HGTLLPLLMLVFGNMTDSFTKAEA--SILPSITNQSGPNSTLIISNSSLEEEMAIYAYYY 117
FhMDR         SGAGFPLNVLIFRSIIDQFIGPSFDASL-----------------------IYGIVKWF 48
FhMDR1        FGVSGPASILIFRVIVNKLISPKITLDN-----------------------IFPLLQWY 83
FhMDR_179     SGTGFPLNILIFRSIINEFTGTNFAASV-----------------------IYDIVGWF 57
               *.  *  :*:*  : : :       .                       :     ::

HsMDR1        SGIGAGVLVAAYIQVSFWCLAAGRQIHKIRKQFFHAIMRQEIGWFDVHDVGELNTRLTDD 178
MmMDR1        TGIGAGVLIVAYIQVSLWCLAAGRQIHKIRQKFFHAIMNQEIGWFDVHDVGELNTRLTDD 177
FhMDR         AVLGACLIVLTFLKSFLFGISAQRQSRRIRVKYFKAVLRQDVPWFDTQSSGSLMNKLTQN 108
FhMDR1        LILGAITFVVSVIQTICFSIVSKRQAQRLRQLYFEAVLNQDVSWFDCQASGSLINNLSDR 143
FhMDR_179     AILGACLAV-------------------------AIMRQDVPWFDEQSTGALMSKLTQN 91
                :**   :                           *::.*:: *** :  * * ..*::

HsMDR1        VSKINEGIGDKIGMFFQSMATFFTGFIVGFTRGWKLTLVILAISPVLGLSAAVWAKILSS 238
MmMDR1        VSKINDGIGDKIGMFFQSITTFLAGFIIGFISGWKLTLVILAVSPLIGLSSALWAKVLTS 237
FhMDR         IDNIEEGIGSKFGEFILNLSGFFCGLIIAFFVGWKLTLVACAMVPFVVTVFLFFGVSMKY 168
FhMDR1        IYDIEQGVGSKLGECIQNASGFISGILIAYIIDWTVALVASASLPLIAFAFSCFGILHKH 203
FhMDR_179     IDNIEEGIGTKFGEFILNISGFFCGIIIAFAVGWKLALVACCMIPVVVIVFALFGFLMKK 151
              : .*::*:* *:*  : . : *: *:::.:  .*.::**  .  *.:      :.   .

HsMDR1        FTDKELLAYAKAGAVAEEVLAAIRTVIAFGGQKKELERYNKNLEEAKRIGIKKAITANIS 298
MmMDR1        FTNKELQAYAKAGAVAEEVLAAIRTVIAFGGQQKELERYNKNLEEAKNVGIKKAITASIS 297
FhMDR         FTMKELDAYSRASAISGEVLSAIRTVMAFGGEKKEFQRYSKELGAAQKMGIIKSAVLGGV 228
FhMDR1        FSQKEKEAYSRASSICGETISAIRTVFAFGGEEKEHKRYTNELKMAEKMGIRKAVAFGGT 263
FhMDR_179     FTLKELQAYAQASGISGEVFSAIRTVVAFGGEKKELQRYSKELGAAQKVGIQKSTALGGV 211
              *: **  **::*..:. *.::*****.****::** :**.::*  *:.:** *: . .

HsMDR1        IGAAFLLIYASYALAFWYGTTLVLSGEYSIGQVLTVFFSVLIGAFSVGQASPSIEAFANA 358
MmMDR1        IGIAYLLVYASYALAFWYGTSLVLSNEYSIGEVLTVFFSILLGTFSIGHLAPNIEAFANA 357
FhMDR         VGAIGLTLFSSAALIFWYGIELIITENYSGGTVVTVFINVVIGSIFLGNALPNMQYFLIA 288
FhMDR1        GGLIGLSMFVCAALVFWFGIKRIVEEQTDAGNLVTVFINIILGSILLGAAASRIPYIMSA 323
FhMDR_179     MGAIGLALFCAAALVFWYGIELLLKEGYRGGSIVTVFVNIIVGSIFLGNALPSMQYFLIA 271
               *   * :: . ** **:*   :     ::     * ::***..:::*:: :*     :  :  *

HsMDR1        RGAAYEIFKIIDNKPSIDSYSKSGHKPDNIKGNLEFRNVHFSYPSRKEVKILKGLNLKVQ 418
MmMDR1        RGAAFEIFKIIDNEPSIDSFSTKGYKPDSIMGNLEFKNVHFNYPSRSEVQILKGLNLKVK 417
FhMDR         MASATEVYGLIDRVPPIDKDR-SGKRIPDFTGNIIFKDVDFAYPTRPDIVVLKKFNLELK 347
FhMDR1        MMSAEEICGTIERKPLIDKNA-KGVILKNFSGEISFENVSFTYPARPDVKVLENFSLHLQ 382
FhMDR_179     MSSAAEVYGTIDRVPPIDKNK-SGKHIPDFAGNIIFKDVDFAYPLRSDAIVLKKFNLELK 330
                :* *:   *:. * **.   .*    .: *:: *.:* * ** * :  :*: :.*.::

HsMDR1        SGQTVALVGNSGCGKSTTVQLMQRLYDPTEGMVSVDGQDIRTINVRFLREIIGVVSQEPV 478
MmMDR1        SGQTVALVGNSGCGKSTTVQLMQRLYDPLEGVVSIDGQDIRTINVRYLREIIGVVSQEPV 477
FhMDR         SGQTVALVGPSGSGKSTIVHMLQRFYDPIGGEIIVEGENIRDLDLKAFRAQLGCVQQEPI 407
FhMDR1        PGTTVALVGPSGSGKSTVVQLLQRFYDTDEGKVLVQGTDVRELDLQHFRQQLGCVQQEPV 442
FhMDR_179     SGQTVALVGPSGSGKSTVVHMLQRFYDPIGGEIIVEGENIRDLDLKAFRAQLGCVQQEPI 390
               * ****** **.**** *:::**:**   * : ::* ::*  :* ::::  :*  :* *.***:

HsMDR1        LFATTIAENIRYGRENVTMDEIEKAVKEANAYDFIMKLPHKFDTLVGERGAQLSGGQKQR 538
MmMDR1        LFATTIAENIRYGREDVTMDEIEKAVKEANAYDFIMKLPHQFDTLVGERGAQLSGGQKQR 537
FhMDR         LFEGTVEDNIRLGKLDATNEEIIEAAKMANAHDFILHLPEGYKTVLAERGAGLSGGQKQR 467
FhMDR1        LFEGTVLENIRLGDMKVSDDEILEAAKLANAHQFITELPDGYNTAVGEGGAGLSGGQKQR 502
FhMDR_179     LFEGTVEDNIRLGKLDATNEEIIEAAKMANAXX--------------------------- 423
              **  *: :*** *  ..: :** :*.* ***

HsMDR1        IAIARALVRNPKILLLDEATSALDTESEAVVQVALDKARKGRTTIVIAHRLSTVRNADVI 598
MmMDR1        IAIARALVRNPKILLLDEATSALDTESEAVVQAALDKAREGRTTIVIAHRLSTVRNADVI 597
FhMDR         IAIARALIRKPKLLLLDEATSALDTRSERVVQEALDQASAGRTVVVVAHRLTTVRNADLI 527
FhMDR1        IAIARALVRNPSLLLLDEATSALDTKSERTVQMALNRASAGRTVLVVAHRLTTIRHADQI 562
FhMDR_179     ------------------------------------------------------------ 423

HsMDR1        AGFDDGVIVEKGNHDELMKEKGIYFKLVTMQTAGNEVELENAADESK---SEIDALEMSS 655
MmMDR1        AGFDGGVIVEQGNHDELMREKGIYFKLVMTQTRGNEIEPGNNAYGSQ---SDTDASELTS 654
FhMDR         MVLDKGVIREKGTHDELVERNGLYAAMLHIQKQTDKNQLGEAEPVD-----DIGLS---- 578
FhMDR1        VVLDKGRICEMGTHEELMLRHGMYASMVKNQEASDDSMNDLEEQEEDEQELNKELIELKS 622
FhMDR_179     XXLDKGVIREKGTHDELVEQNGLYAAMLRRQKQTEKQIPEEGETEV-----ETELEGLHN 478
                :* * * * *.*:**: .:*:*  ::  *   :.              :
```

```
HsMDR1          NDSRSSLIRKRSTRRSVRGSQ--AQDRKLSTKEALDES---IPPVSFWRIMKLNLTEWPY 710
MmMDR1          EESKSPLIRR-SIYRSVHRKQ--DQERRLSMKEAVDED---VPLVSFWRILNLNLSEWPY 708
FhMDR           --------KKTNLRNGSNGAMWTL----------ADDDIFTFKRSPIFRILKMNRTELGY 620
FhMDR1          -----GNIPNTDDVYSIVGSLYSVSTSDLGTKSKVTNSLKRLKNSTAMRLIRLNRPELPF 677
FhMDR_179       -----PD-EGTKTSEGLVSAVWKI----------ADNDMRGIRNSPIFRILKMNRPELGF 522
                           .   .                    :.   .      *::.:*  *  :

HsMDR1          FVVGVFCAIINGGLQPAFAIIFSKIIGVFTRIDDPETKRQNSNLFSLLFLALGIISFITF 770
MmMDR1          LLVGVLCAVINGCIQPVFAIVFSRIVGVFSRDDDHETKRQNCNLFSLFFLVMGLISFVTY 768
FhMDR           IIGGCVASLIAGAVQPSFALLYSQMFQIFTYANDPEKMRFQVSIFAALMVFLGCLRFLGM 680
FhMDR1          LLAGCLCSTLSGACQPIFGILYSEVYAIFQMD--KSQMQDRVNVISAAMVGVGVARLLLG 735
FhMDR_179       IIGGCIVSVISGASQTAFSLVYSEMFQIFTYINEPDKMRSQVGLFAGLMVLMGVLRFLSM 582
                :: * . : : *  *  *.:::*.:  :*      .  : . .:::  :: :*   ::

HsMDR1          FLQGFTFGKAGEILTKRLRYMVFRSMLRQDVSWFDDPKNTTGALTTRLANDAAQVKGAIG 830
MmMDR1          FFQGFTFGKAGEILTKRVRYMVFKSMLRQDISWFDDHKNSTGSLTTRLASDASSVKGAMG 828
FhMDR           LGQGFFFGVSGERLTRRVRGMLFQAIMRQEIGWFDRTENQPGTLTAVLATEASKMKSLSG 740
FhMDR1          FGQGYFFGVSGEKLIRRMRSTVFKSILRQEVGWFDRSENQAGSLTALLATEATKLSQITG 795
FhMDR_179       LGQGFFFGVSGERLTRRVRCKLFEAVMKQEIGWFDRTENQPGVLTVLLATEASKLKSISG 642
                : **: ** :** * :*:*  :*.::::*::.***  :*  * **. **.:*:.:.   *

HsMDR1          SRLAVITQNIANLGTGIIISFIYGWQLTLLLLAIVPIIAIAGVVEMKMLSGQALKDKKEL 890
MmMDR1          ARLAVVTQNVANLGTGVILSLVYGWQLTLLLVVIIPLIVLGGIIEMKLLSGQALKDKKQL 888
FhMDR           SQLGFIIEAFALVTMSLVIAFIYSWQLTLLMLAFYPMFILSGMLQIKRIAGEGSKE--TD 798
FhMDR1          TRLGSLCEVIMLIVISLSVAFVFSWQLTLLALAFFPFLAIGTYLQHRVFGQSNNEN--AD 853
FhMDR_179       AQLGYIVEAIVMVVMSLVITFIYSWQLTLLMLAFYPLFVLTGMIQIQRMSGGNSKK--TD 700
                ::*. : : .  :  .: :::::.****** :.: *:: :   :: : :.    :.

HsMDR1          EGSGKIATEAIENFRTVVSLTQEQKFEHMYAQSLQVPYRNSLRKAHIFGITFSFTQAMMY 950
MmMDR1          EISGKIATEAIENFRTIVSLTREQKFETMYAQSLQVPYRNAMKKAHVFGITFSFTQAMMY 948
FhMDR           GPSTQLAQEAISSDRTVFTLNLEDYFQNRFEDLLMNKKLSNLKQTILFAVLFSLTQAGPM 858
FhMDR1          NTSVRIAQEVFRAQRTVTSFSLEKHFCRRFERSVSEYMRSQLRNSITFGILYAFAMVIPT 913
FhMDR_179       ITSTRVAQEAIGSDRTLFTLTLEDYFCSRYENSLLHNKKSNLKKIAVYAVLFSLTQAGPM 760
                  * ::* *.:   **: ::. *. *   :   :     . :::   :.: :::: .

HsMDR1          FSYAGCFRFGAYLVAHKLMSFEDVLLVFSAVVFGAMAVGQVSSFAPDYAKAKISAAHIIM 1010
MmMDR1          FSYAACFRFGAYLVAQQLMTFENVMLVFSAVVFGAMAAGNTSSFAPDYAKAKVSASHIIR 1008
FhMDR           FCFAAAFTLGAFLVEHKYIEMLAVFRVFAVLNMSAQSLGRTASIGPETQRAREASKIILK 918
FhMDR1          LLFCALFALGTHLIETEVIDMTALFRVFLVFSMSSQGIGHAVSASPDASAAIISARRMLF 973
FhMDR_179       FCFAAAFALGAYLVGRHDIQMLAVFRVFSVQNLTAQSLGRTASYGPESLRAQNASKAILQ 820
                : :.. * :*:.*:  . : :  :: ** .  : : . *.. * .*:   *  ::  ::

HsMDR1          IIEKTPLIDSYSTEGLM-PNTLEGNVTFGEVVFNYPTRPDIPVLQGLSLEVKKGQTLALV 1069
MmMDR1          IIEKTPEIDSYSTEGLK-PTLLEGNVKFNGVQFNYPTRPNIPVLQGLSLEVKKGQTLALV 1067
FhMDR           TLDRTPSIRTD--DGLVPREPFRGNMEFKHVYFSYPTRKTISVLKKFSHAVNSGETVALV 976
FhMDR1          IMDRIPKIQTN--VGDVPKSSLKGHIEFKNVHFRYPTRPDVRVLKNFSHTISPGQSVAVV 1031
FhMDR_179       LLDRNPSIRTD--EGSVPQEPFKGHVEFKRIYFSYPTRKTLVVLKNFSHVVNPGETVALV 878
                 ::: * * :    *      :.*:: *  : * ****  : **: :*  :. *:::*:*

HsMDR1          GSSGCGKSTVVQLLERFYDPLAG-----KVLLDGKEIKRLNVQWLRAHLGIVSQEPILFD 1124
MmMDR1          GSSGCGKSTVVQLLERFYDPMAG-----SVFLDGKEIKQLNVQWLRAHLGIVSQEPILFD 1122
FhMDR           GESGCGKSTLLQLVQRFYDPHDFGS-DSGIFFDGHNIRQLAPAWVRRQIGIVSQEPNLFD 1035
FhMDR1          GQSGCGKSTLIQLLLRFYDPSNQKLDQVGIFFDGMNLRNLAPSWIRHQIGLVSQEPVLFN 1091
FhMDR_179       GESGCGKSTLLQLVQRFYDPISVGS-DSGIFFDGHNIRQLAPAWVRRQIGIVSQEPNLFD 937
                *.*******::**: *****         :::** :::.*   *:* ::*:***** **:

HsMDR1          CSIAENIAYGDNSRVVSQEEIVRAAKEANIHAFIESLPNKYSTKVGDKGTQLSGGQKQRI 1184
MmMDR1          CSIAENIAYGDNSRAVSHEEIVRAAKEANIHQFIDSLPDKYNTRVGDKGTQLSGGQKQRI 1182
FhMDR           MSIRDNIAYGDNSREVTMDEIMEAARQANVHEFITTLPEGYDTSVGQKGSHLSGGQKQRV 1095
FhMDR1          MSISDNIAYGDNTRVLSMDEIVEAARLANIHEFIASLPNGYETLAGEGGSQLSGGQKQRI 1151
FhMDR_179       MSIRDNIAYGDNSREVTIDEIIEAARQANIHEFITTLPEGYETSAGQKGSRLSGGQKQRV 997
                 ** :*******:* :: :**:.**: **:* ** :**: *.* .*: *::********:

HsMDR1          AIARALVRQPHILLLDEATSALDTESEKVVQEALDKAREGRTCIVIAHRLSTIQNADLIV 1244
MmMDR1          AIARALVRQPHILLLDEATSALDTESEKVVQEALDKAREGRTCIVIAHRLSTIQNADLIV 1242
FhMDR           AIARALLRKPILLLLDEATSALDVESERVVQEALDSAMGSRTCLMVAHRLSTVESADLIV 1155
FhMDR1          AIARALLRKPALLLLDEATSSLDSENERLVQSALDAARASRTAIVIAHRLTTVENSDMIV 1211
FhMDR_179       AIARALLRKPILLLLDEATSALDVESERVVQDALDSAMGSRTCLVVAHRLSTVENADLIV 1057
                ******:*:* :********:** *.*::**.*** *  .**.:::****:*::.:*:**

HsMDR1          VFQNGRVKEHGTHQQLLAQKGIYFSMVSVQAGTKRQ    1280
MmMDR1          VIENGKVKEHGTHQQLLAQKGIYFSMVQAGAK--RS    1276
FhMDR           VLENGRKIEAGAPEALLNAKGAFYTLHHTENAATY-    1190
FhMDR1          VIQDGKKIEEGKPSELLEARGAFYALHHVQKESQ--    1245
FhMDR_179       VLQNGQKIEVGAPEALINAKGAFYALHHTENATA--    1091
                *:::*:  * *  . *:  :* ::::  .
```

B

Percent Identity Matrix

	HsMDR1	MmMDR1	FhMDR	FhMDR1	FhMDR_179
1: HsMDR1	100.00	80.53	44.15	39.40	40.98
2: MmMDR1	80.53	100.00	42.98	39.07	41.64
3: FhMDR	**44.15**	**42.98**	100.00	51.90	77.51
4: FhMDR1	39.40	39.07	51.90	100.00	50.51
5: FhMDR_179	40.98	41.64	77.51	50.51	100.00

Supplementary Figure S6 Multiple alignment of amino acid sequences of multidrug resistance proteins (MDR). (A) Amino acid sequences of MDR orthologues of *Homo sapiens* (Hs), *Mus musculus* (Ms) and *Fasciola heptica* (Fh) were aligned using CLUSTAL OMEGA. Yellow boxes: ABC membrane domains. Blue boxes: AAA, ATPase domains. Accession numbers: HsMDR1, NP_001335873.1; MmMDR1, NP_035205.1; FhMDR, maker-scaffold10x_1211_pilon-augustus-gene-0.53; FhMDR1, maker-scaffold10x_794_pilon-augustus-gene-0.66; FhMDR_179, maker-scaffold10x_157_pilon-snap-gene-0.179. **(B)** Percent Identity Matrix generated from the alignment shows the percentage of identical amino acids between MDR proteins of the various species (in %). Among the putative *F. hepatica* orthologues, FhMDR has highest identity to known mammalian MDR1 orthologues (42.98% and 44.15%).

3 Supplementary Videos

Supplementary Video S1 *Fasciola hepatica* adult fluke depicts normal motility after 72 h treatment with DMSO as control.

Supplementary Video S2 *Fasciola hepatica* adult fluke with a motility score of 1 (minimal activity) after 72 h treatment with 150 µM imatinib.
